# Invasive Meningococcal Disease and Meningococcal Serogroup B Vaccination in Adults and Their Offspring: Knowledge, Attitudes, and Practices in Italy (2019)

**DOI:** 10.3390/vaccines11030508

**Published:** 2023-02-22

**Authors:** Matteo Riccò, Milena Pia Cerviere, Federico Marchesi, Marco Bottazzoli

**Affiliations:** 1 Occupational Health and Safety Service on the Workplace/Servizio di Prevenzione e Sicurezza Ambienti di Lavoro (SPSAL), Department of Public Health, AUSL–IRCCS di Reggio Emilia, 42122 Reggio Emilia, Italy; 2 Università Cattolica del Sacro Cuore, 00168 Rome, Italy; 3 Department of Medicine and Surgery, University of Parma, 43126 Parma, Italy; 4 Department of Otorhinolaryngology, APSS Trento, 31223 Trento, Italy

**Keywords:** vaccine preventable diseases, meningitis, meningococcus, knowledge, risk perception

## Abstract

Despite its effectiveness in preventing invasive meningococcal disease (IMD), pediatric uptake of recombinant meningococcal vaccination for serogroup B meningitis (MenB) is low in Italy. This study aimed to investigate knowledge, attitudes, and practice (KAP) about IMD and the vaccine uptake for MenB from July to December 2019, in a sample collected from a series of local Facebook discussion groups from the provinces of Parma and Reggio Emilia (North-Eastern Italy; 337,104 registered users). A self-administered anonymous web-based questionnaire was used to collect demographics, knowledge status, perceived risk for contracting meningitis, attitude towards the utility of meningococcal vaccine, and willingness to receive/perform MenB vaccine in their offspring. In total, 541 parents returned a fully completed questionnaire (response rate of 1.6% of potential recipients), with a mean age of 39.2 years ± 6.3 (78.1% females). Meningococcal infection was identified as severe or highly severe by most participants (88.9%), while it was recognized as being frequent/highly frequent in the general population by 18.6% of respondents. The overall knowledge status was unsatisfactory (57.6% ± 33.6 of correct answers to the knowledge test). Even though 63.4% of participants were somewhat favorable to MenB/MenC vaccines, offspring’s vaccination towards MenB was reported by only 38.7% of participants. In a binary logistic regression model, the male gender of respondents (adjusted odds ratio [aOR] 3.184, 95% confidence interval [95%CI] 1.772 to 5.721), living in a municipality >15,000 inhabitants (aOR 1.675, 95%CI 1.051 to 2.668), reporting a favorable attitude on meningococcus B vaccine (aOR 12.472, 95%CI 3.030 to 51.338), having been vaccinated against serogroup B (aOR 5.624, 95%CI 1.936 to 16.337) and/or serogroup C (aOR 2.652, 95%CI 1.442 to 4.872), and having previously vaccinated their offspring against serogroup C meningococcus (aOR 6.585, 95%CI 3.648 to 11.888) were characterized as positive effectors of offspring’s vaccination. On the contrary, having a higher risk perception on vaccines was identified as the only negative effector (aOR 0.429, 95%CI 0.241 to 0.765). Our results hint towards extensive knowledge gaps on IMD and preventive interventions in the general population, suggesting that a positive attitude towards vaccines and vaccinations could be identified as the main effector also for MenB acceptance. Interventions in the general population aimed at improving confidence, compliance, and acknowledgment of the collective responsibility, as well as preventing actual constraints and the sharing of false beliefs on infectious diseases and their preventive measures, could therefore increase vaccination acceptance in both targeted individuals and their offspring.

## 1. Introduction

Invasive meningococcal disease (IMD) is a condition caused by the bacterium *Neisseria meningitidis* or meningococcus, a Gram-negative diplococcus, which colonizes the pharynx and upper respiratory tract [1,2]. IMD is a major cause of bacterial meningitis and septicemia, with a high case fatality ratio and similarly high rates of long-term sequelae [3,4,5]. Incidence rates of IMD vary geographically and over time, but their occurrence is usually higher among infants, followed by a second peak in adolescents and young adults [6,7,8,9].

To date, 12 serogroups of meningococcus have been identified based on bacterial capsular polysaccharide [6,8,10,11,12]. Of them, serogroups A, B, C, W, X, and Y cause virtually all human cases [1,13,14], with serogroup B having been characterized as the most common in European countries [1,2,8,13,14]. For instance, between 2004 and 2014, serogroup B accounted for 74% of 49,269 cases of IMD with a known serogroup [4]. Even after the introduction of meningococcal serogroup B vaccine (MenB) in 2014, in 2018, approximately 51% of 3316 confirmed cases of IMD from the 30 European Union/European Economic Area (EU/EEA) member states (overall notification rate of 0.6 cases per 100,000) still belonged to serogroup B, followed by groups W, C, and Y (18%, 15%, and 12%, respectively) [15]. 

Since 2014, incidence rates of serogroup B IMD have slowly but steadily declined [1,8,15,16], particularly in countries where high levels of vaccination coverage have been achieved [15]. For example, shortly before the inception of the MenB vaccination campaign, Lithuania had experienced very high rates of IMD (2.56 per 100,000 persons in 2013), but it dropped to 1.1 per 100,000 in 2018 [15]: in this timeframe, the incidence of serogroup B IMD declined from 1.62 per 100,000 in 2012 to 0.46 in 2018. Contrarily to the vaccine against serogroup C (MenC), MenB does not guarantee any effect on meningococcal carriage, only providing direct protection against IMD [17,18,19,20]. As a consequence, high vaccination rates among high-risk groups (i.e., children and adolescents, as well as adults affected by significant comorbidities) are instrumental in reducing the risk for IMD [19,21,22,23]. 

With official notification rates ranging between 0.52 cases per 100,000 in 2004 and 0.28 cases in 2018 [15], Italy has been extensively acknowledged as being a relatively low risk for IMD, and is largely below the threshold identified by WHO for large-scale meningococcal vaccine programs [24]. Still, Italian Health Authorities have actively implemented childhood vaccination against MenC since the end of the 1990s, and two MenB vaccines have been implemented in the Italian “lifetime immunization schedule” since 2014, with both recommended and publicly funded by the National Vaccination Prevention Plan 2017–2019 (NVPP) [23,25,26]. More precisely, Italian Medicines Agency (AIFA) has authorized the use of a quadrivalent recombinant vaccine developed through “reverse vaccinology” (i.e., preparation of the vaccine starting from the identification of genes encoding antigenic proteins), or 4CMenB, among infants >2 months since 2014, and a bivalent recombinant vaccine (MenB-FHbp) in children >10 years of age since 2017 [23]. Despite considerable efforts, vaccination rates for MenB have remained suboptimal due to a series of factors, ranging from the heterogeneity in the vaccination policies in terms of official recommendations and the actual costs of the vaccine [19,27,28,29], limited effectiveness of the sanctioning system, low-risk perception of infectious diseases, confidence in the availability of antimicrobial therapies, scientific misinformation, and the re-emergence of anti-vaccination movements [25,30,31]. More precisely, while all children born after 2015 were offered MenB vaccine totally at no cost to their parents, parents of individuals born before 2015 and not affected by risk factors for IMD may be requested to partially or even totally contribute to the costs of the vaccine [19]. Since nearly all vaccination programs were affected by various degrees of vaccine hesitancy (i.e., delay in acceptance or refusal of vaccines despite the availability of vaccination services) [32,33,34], with resulting failures in obtaining targeted vaccination rates, in 2017, the Italian government prompted the introduction of Law No. 119, which extended mandatory vaccinations for children up to 16 years of age and for unaccompanied foreign minors [31]. Interestingly, meningococcal vaccines (i.e., MenB, MenC, and the tetravalent MenACWY) were not included in this list, but a recent report stressed how vaccination rates for MenB have significantly increased between 2016 and 2019 (+54.3%). 

In this regard, the SARS-CoV-2 pandemic has represented a substantial game-changer in both the epidemiology and prevention of IMD [1]. On the one hand, non-pharmaceutical interventions (NPI: interventions other than immunization or pharmaceutical therapies that communities can take to help slow the spread of illnesses that aim to prevent and/or control the pathogen’s transmission in the community) [35,36] have contributed to a substantial decline in IMD cases relative to previous years, not only at EU level (0.26 cases per 100,000 in 2020 vs. 0.57 per 100,000 in 2019), but also in Italy (0.12 cases per 100,000 in 2020 vs. 0.31 per 100,000 in 2019), despite some regional heterogeneities [37]. On the other hand, vaccination rates for meningococcal vaccines have either slowed or even declined in many countries. According to available data, up to 50% of Italian parents declared to either have delayed or canceled a scheduled meningococcal vaccination appointment for their children during the pandemic [1], with a reduction in MenB rates estimated to be −2.68% in 2020 vs. 2019, and a subsequent recovery in 2021 (+13.38% in 2021 vs. 2020) [38,39].

Shortly before the inception of the SARS-CoV-2 pandemic, we designed a cross-sectional study targeting parents of children aged 14 years or less on their knowledge (i.e., awareness of official recommendations), attitudes (i.e., propensity towards vaccinations), and practices (i.e., actual uptake of vaccination; collectively KAP) on MenB. Our primary objective was to investigate their adherence to the official schedule for MenB, investigating their respective KAP, and specifically focusing on vaccine hesitancy and its main drivers. By providing a sort of “historical” picture of the acceptance of vaccination policies, as perceived shortly before an unprecedented health event (i.e., the COVID-19 pandemic), our study could share a valuable contribution in the design of appropriate interventions for improving vaccine acceptance for MenB.

## 2. Materials and Methods

### 2.1. Study Design and Sample Size

The present study was designed as a cross-sectional questionnaire-based study (see STROBE Checklist as Supplementary File S1), and was performed between 1 July 2019 and 31 December 2019, targeting 89 Facebook discussion groups from 44 municipalities from the province of Parma and 42 from the province of Reggio Emilia (Emilia Romagna Region), with an overall population of 454,873 and 529,609 inhabitants (census 2020), respectively, and a total surface area of 5738.74 km^2^. Group pages had approximately 132,784 (Parma) and 205,330 (Reggio Emilia) unique members, equal to 34.3% of the target population (29.2% for Parma, 38.8% for Reggio Emilia), but no information could be obtained regarding cross-inscriptions, nor on how many of these members were actively using Facebook.

As childhood official vaccination rates in the Emilia Romagna Region for MenB were estimated to be 19.16% at 24 months and 21.12% at 36 months, by assuming a Type I error of 5% (0.05) and power of 95%, the minimum sample size for each province was calculated as follows:

N = 1.96^2^ × 0.8 × (1 − 0.8)/0.05^2^ = 3.8416 × 0.8 × 0.2/0.0025 = 246 (1)

Therefore, by assuming a minimum sample size of 246 respondents for each province, a total of 492 respondents were targeted for the present study.

In order to post the study invitation, the chief researcher (MR) contacted the administrators and requested their preventive authorization to post a link to the questionnaire. The link included a short description of the aims of the survey and led to a web page including the full study information and the informed consent (Supplementary File S2). Individuals agreeing to participate in the survey were able to access the web survey (Google Forms; Google LLC; Menlo Park, California, CA, USA). 

### 2.2. Instruments

Subjects who gave their consent received the link to a structured Google Forms survey including three preliminary questions corresponding to the inclusion criteria: (1) living in the province of Parma or Reggio Emilia; (2) being aged 18 years or older; (3) having any child aged 14 years or less. Participants fulfilling criteria 1 and 2 were allowed to complete the questionnaire, with the exclusion of the section specifically dealing with vaccination in offspring. On the contrary, if a potential participant was found not to match inclusion criteria 1 and/or 2, the survey was stopped, with the potential participant unable to provide answers to the following items of the questionnaire. The survey was anonymous and no personal data, such as name, IP address, email address, or personal information unnecessary to the survey, was requested, saved, or tracked. No monetary or other compensation was offered to the participants. 

The questionnaire included a series of items previously developed for KAP inquiries [40,41,42,43,44,45,46,47,48], divided into the following areas of inquiry:

1. Demographic characteristics of the participants. Age, sex, professional qualifications, education level, household characteristics, whether they had any background (occupational or educational) in healthcare settings.
2. Knowledge Test. Participants received a knowledge test, containing a total of 18 true–false statements, such as “Measles vaccine may elicit autism” (false), covering some typical misconceptions on vaccines and immunizations (items Q6 to Q18) and, more specifically, targeting IMD and meningococcal vaccines (items Q1 to Q5) [4,7,9,45,46,47,48,49]. A summary score (General Knowledge Score or GKS) was calculated as follows: when the participant provided a correct answer, +1 was added to a sum score, whereas a wrong indication or a missing answer added 0 to the sum score.
3. Risk Perception. Risk perception is a significant component of attitude and has been defined as a function of the perceived probability of an event and its expected consequences, being assessed as the mathematical product of subjective probability and disease severity [43,50,51,52]. Consequently, we asked the participants about their perceived probability of (a) meningococcal infections (E^inf^), (b) vaccine-related adverse effects (E^vac)^, and whether they perceived the severity of the (c) natural infection (C^inf^) and (d) vaccine-related adverse effects (C^vac^). In order to summarize the results, we used a fully labelled 5-point scale (“almost zero”, “low or rather low”, “moderate”, “high or rather high”, “very high”). Two risk perception scores (RPS) were eventually obtained through the formulas:

RPS^inf^ = E^inf^ × C^inf^ (2)

RPS^vac =^ E^vac^ × C^vac^ (3)

where:

RPS^inf^ = Risk Perception Score for natural infections;

RPS^vac^ = Risk Perception Score for vaccinations;

E^inf^ = perceived probability of contracting a meningococcal infection;

E^vac^ = perceived probability of developing complications after the delivery of vaccine;

C^inf^ = perceived severity of meningococcal infection;

E^vac^ = perceived severity of vaccine side effects.

4. Attitudes toward vaccinations. Participants’ attitude towards vaccines and immunizations was asked both in general and more specifically towards MenB/MenC, through a 5-point Likert scale (“strongly against”, “against”, “neutral/no opinion”, “favorable”, “strongly favorable”). In the analyses, the results were dichotomized as somehow favorable (“strongly favorable” and “favorable”) vs. somehow against/neutral (“neutral”, “against”, “strongly against”): in both cases, a selected set of declarative sentences was presented to the participants regarding their reason to respectively accept immunizations and more specifically meningococcal vaccines (i.e., “to avoid getting vaccine preventable diseases (VPDs)”, “to avoid transmitting VPDs”, “to avoid complications of VPDs”, “to avoid VPDs in subjects who cannot be vaccinated”) or instead refuse them (e.g., “to avoid shots/medications”, “uselessness”, “fear of side effects”, “religious/ethical reasons”, etc.). Eventually, participants were asked to summarize their perceived trust towards vaccines and healthcare providers through a 5-point Likert scale (“scarce” to “optimal”).
5. Practices. All participants were asked whether they had been previously vaccinated for serogroup B meningitis. Participants having children 14 years old or younger were then asked whether their offspring had previously received MenB vaccine, or whether immunization was planned to be performed in the next six months.

### 2.3. Ethical Considerations

This study was conducted according to the guidelines of the Declaration of Helsinki. We opted for an anonymous, observational design, and deliberately limited the extent of retrieved information. As some participants could develop stress, anxiety, and even panic about this disease through an inappropriate understanding of its features, we provided the correct answer for all items of the knowledge test as a plain text, which was made available to participants after the completion of the questionnaire. As stated in the informed consent, we specifically guaranteed the confidentiality of all information reported by study participants. Moreover, the design of the questionnaire deliberately avoided the recall of clinical data, and limited the collection of personal data to quite general information. In this regard, participants were also guaranteed that retrieved data would be stored only for the time required by the data analysis. As participants cannot be individually identified through the presented material and retrieved demographic data, and the present survey did not include clinical data about participants, the present study reasonably caused no harm or stigma to participants. Therefore, this study did not configure itself as a clinical trial and no preliminary evaluation by an Ethical Committee was statutorily required, according to Italian law (Gazzetta Ufficiale No. 76, dated 31 March 2008) (Supplementary File S4).

### 2.4. Data Analysis

As a preliminary step, two independent researchers preventively checked the accuracy of collected data, with incomplete questionnaires excluded from the analyses. Cumulative scores (RPS, GKS) were then normalized to percentage values, with the latter also dichotomized by median value as “high” and “low” groups. All continuous variables were expressed as mean ± standard deviation. Their distributions were preventively assessed by means of the D’Agostino–Pearson K2 test, and were compared through a Student’s t-test for unpaired data or ANOVA when normally distributed (i.e., K2 test *p* value > 0.100), or through a Mann–Whitney or Kruskal–Wallis test when not normally distributed (i.e., K2 test *p* value < 0.100). The correlation between continuous variables was assessed by the calculation of Pearson’s correlation coefficient or through Spearman’s rank test according to the distribution of the data. Next, categorical variables were reported as percentage values, and their distribution in respect of the outcome variable represented by a somewhat positive attitude towards MenB was initially analyzed through a chi-squared test. Eventually, all variables during univariate analysis that were significantly (*p* < 0.05) associated with having vaccinated offspring against serogroup B meningococcus were included in a stepwise binary logistic regression analysis model in order to calculate their multivariate odds ratios (aOR) and their respective 95% confidence intervals (95%CI). All statistical analyses were performed by means of IBM SPSS Statistics 26.0 for Macintosh (IBM Corp. Armonk, NY, USA).

## 3. Results

### 3.1. Demographics of Participants

**Figure 1:**
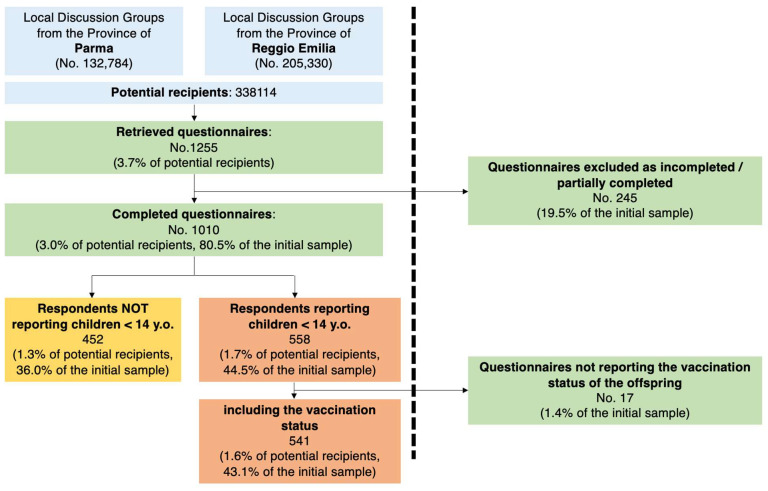
Flow chart of included questionnaires.

The convenience sample eventually included a total of 1255 questionnaires (3.7% of the potentially eligible population). Of them, 245 were excluded from further analyses as they lacked some demographic features. Of the 1010 completed questionnaires, 558 (55.2%) fulfilled all inclusion criteria as they reported at least one child <14 years old, compared to 452 (44.8%) participants not reporting any children (Figure 1). Of these, 541 (97.0%) included detailed information on the vaccination status of their offspring.

Detailed characteristics of the sample as a whole are reported in Appendix A Table A1. Focusing on the 558 participants fulfilling inclusion criteria (Table 1), the majority of respondents were of the female gender (78.1%). Mean age was 39.2 years ± 6.3 (47.1% were aged 40 years or more). The majority (65.2%) of participants had a university-level degree and, when dealing with residency, the majority lived in municipalities with 15,000 inhabitants or more (59.6%), in households of 3 persons or more (97.8%). Interestingly, 56.0% of participants reportedly belonged to a household of 4 persons or more and, in 13.3% of cases, it included at least one subject aged 65 years or older. Eventually, 26.7% of participants had a background in healthcare settings. 

In fact, the sub-group of participants that had parented at least one child aged < 14 years, and were therefore included in further analyses, was characterized by a higher proportion of individuals of the female gender (78.1% vs. 67.7%, *p* < 0.001), a substantially higher number of individuals holding a university-level degree (65.2% vs. 57.5%, *p* = 0.030), and a quite different composition of their household. Not only did this sub-group include 3 or 4 persons more often than those participants not reporting children < 14 years (41.2% and 45.2% vs. 22.1% and 23.5%, respectively, *p* < 0.001), but a lower proportion of respondents acknowledged living with individuals aged 65 years of more (13.3% vs. 20.8%, *p* = 0.001). Interestingly, the share of participants with an occupational background in healthcare settings was higher among individuals not parenting children < 14 years than among those who were included in further analyses (35.6% vs. 26.7%, *p* = 0.002).

### 3.2. Knowledge Test

**Figure 2:**
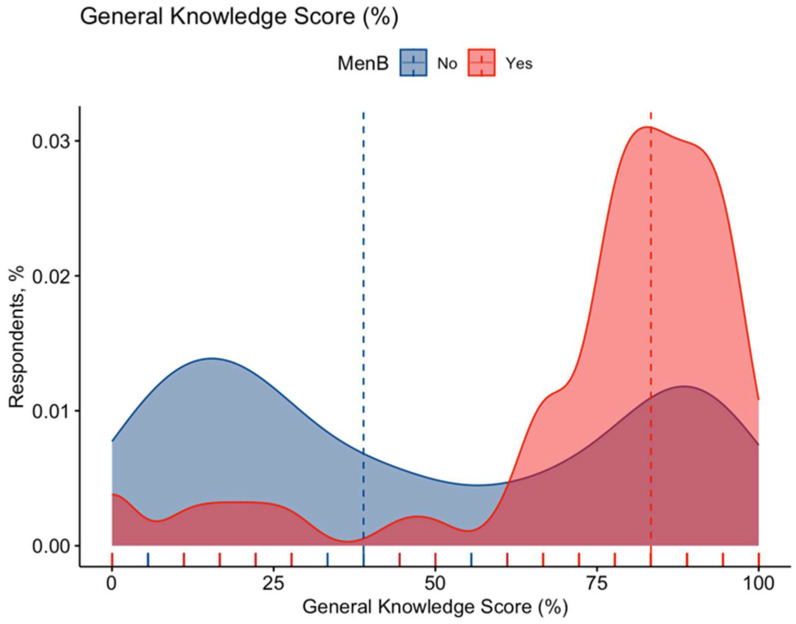
Density plots on General Knowledge Score (GKS) for participants fulfilling all inclusion criteria (No. = 558, 55.2% of the original sample), classified as having or not having received MenB vaccine. Distributions did not pass the normality check (D’Agostino–Pearson K2 = 8486.0; *p* < 0.001); individuals having been vaccinated against meningococcus serogroup B (MenB) reported substantially higher estimates (74.7% ± 24.6 vs. 47.5% ± 34.5; Mann–Whitney U 48,958.0, *p* < 0.001).

**Figure A2:**
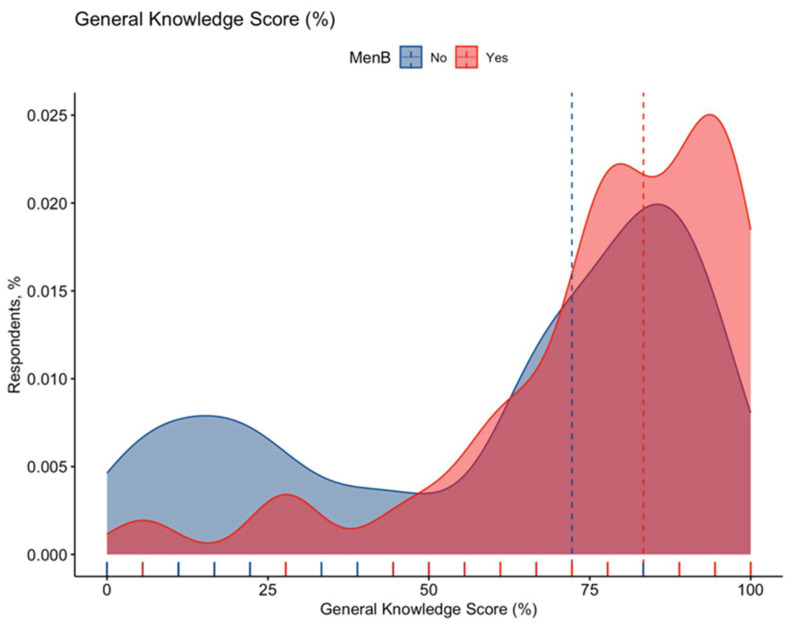
Density plots on General Knowledge Score (GKS) for the sample as a whole (No. = 1010, 100% of the sample), classified as having or not having received the vaccine against serogroup B meningococcus. Overall distribution did not pass the normality check (D’Agostino–Pearson K2 = 149.5); individuals having been vaccinated with MenB reported higher estimates (77.6% ± 21.5 vs. 61.4% ± 30.8; Mann–Whitney U 84,557.5, *p* < 0.001).

The internal consistency coefficient calculated on the knowledge test amounted to Cronbach’s alpha = 0.938, suggesting an acceptable reliability of the Italian version of the questionnaire. After percentage normalization, there was an unsatisfactory GKS estimate of 57.6% ± 33.6 (actual range 0.0% to 100%, median 72.2%), whose distribution of the cumulative score did not pass the normality check (D’Agostino–Pearson K2 = 8486.0; *p* < 0.001) (Figure 2; Table A2). Similar estimates for the sample as a whole are reported in Figure A2.

Detailed results of the knowledge test are reported in Table 2 (see Table A3 for the complete sample). The main knowledge gaps can be identified in specific features of IMD and meningococcal vaccines. In fact, less than half of respondents (42.5%) had any knowledge that meningococcus causes half of all bacterial meningitis, approximately 40–50% of all bacterial meningitis in children aged ≥2 years, and more than 70% in children aged ≥5 years (Q01), and that MenB vaccine may be associated with other vaccinations and vaccine formulates (Q05; 40.7%). Even general knowledge issues on vaccines were affected by substantial misunderstandings, as only 37.6% of respondents acknowledged the lack of potential side effects after receiving seasonal flu vaccines (Q08).

### 3.3. Risk Perception

As reported in Table 3, nearly all respondents (88.9%) perceived meningococcus as a severe or even potentially lethal pathogen, while its occurrence was reported as high or very high in the general population by less than one-fifth of participants (i.e., 18.6%). 

**Figure 3:**
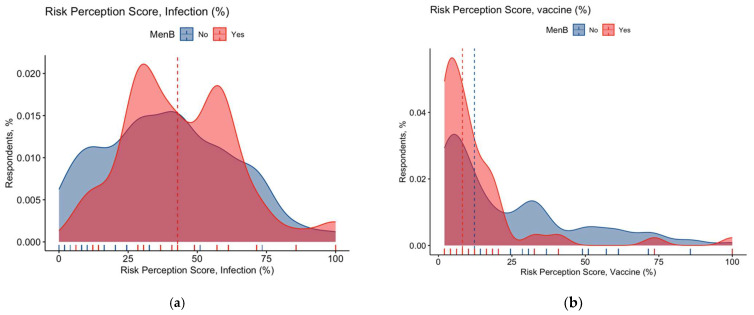
Density plots for Risk Perception Score (RPS) on Neisseria meningitis B serogroup natural infections (**a**) and vaccines (MenB) (**b**) in participants reporting any son/daughter (No. = 558, 55.2% of the original sample). All cumulative scores were substantially skewed (K2 = 11.36, *p* = 0.003 for RPS on natural infection; K2 = 72.75, *p* < 0.001 for RPS on vaccines). When cumulative scores were classified as having or not having received MenB, the perceived RPS for natural infection was higher among vaccinated than among unvaccinated (44.8% ± 20.5 vs. 39.8% ± 23.7; Mann–Whitney (M–W) U 39,307.0, *p* = 0.017), while RPS for the vaccine was conversely lower among vaccinated than among unvaccinated (16.8% ± 21.3 vs. 32.9% ± 26.2; M–W U 19,615.0, *p* < 0.001).

**Figure A3:**
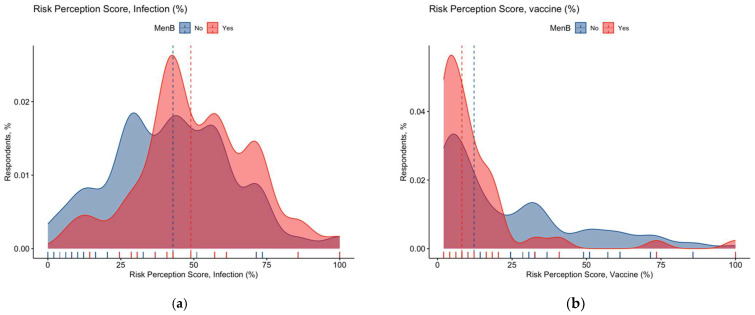
Density plots for Risk Perception Score (RPS) on Neisseria meningitis B serogroup (MenB) natural infections (**a**) and vaccines (**b**) for the whole of the sample (i.e., 1010 respondents). All cumulative scores were substantially skewed (K2 = 6.830, *p* = 0.033 for RPS on natural infections; K2 = 219.7, *p* < 0.001 for RPS on vaccines). When cumulative scores were classified as having or not having received MenB vaccine, perceived RPS was higher among vaccinated than among unvaccinated (50.9% ± 19.2 vs. 42.6% ± 21.1; Mann–Whitney (M–W) U 79,134.0, *p* < 0.001), while RPS was conversely lower among vaccinated than among unvaccinated (12.8% ± 17.6 vs. 23.4% ± 23.6; M–W U 47,625.0, *p* < 0.001).

When focusing on the risk perception for vaccines against meningococcus, 28.5% of all respondents acknowledged meningococcal vaccines as being associated with either severe or very severe side effects, while 26.3% reported side effects as either severe or highly severe. The corresponding RPS accounted for 41.6% ± 22.3 for meningococcal natural infection and 26.2% ± 25.8 for meningococcal vaccines (Figure 3 and Figure A3). 

### 3.4. Attitudes and Practices towards Meningococcal Vaccines

The majority of respondents exhibited high or very high confidence in vaccines (in general, 59.0%), and particularly on MenB (64.2%) (Table 3), while their attitude towards the acceptability of vaccines was similarly high (65.8% for vaccines in general and 63.4% for meningococcal vaccines). When dealing with their practices, i.e., having been or having been not vaccinated against meningococcus, 21.5% of respondents were reportedly vaccinated against serogroup C meningococcus, while 9.5% were vaccinated against serogroup B meningococcus.

When focusing on the perceived drivers for vaccinating themselves and their offspring against serogroup B meningococcus (Table 4; see also Table A4 for the sample as a whole), the most frequently reported reasons were identified as: avoiding the complications of meningitis (78.2%) and being protected against as many infectious diseases as possible (72.7%), followed by avoiding bacterial meningitis (70.4%), acknowledging meningitis as a severe disease (64.4%), protecting those who cannot be vaccinated (62.2%), being protected against meningitis (56.0%), and avoiding the transmission of meningitis (53.7%). On the contrary, less than half of respondents reported the inclusion of meningococcus B vaccine into the National Vaccination Plan (35.6%) and the suggestion of a pediatrician (20.4%) as a main driver, while only 7.4% reported the previous infection of acquaintances, friends, or relatives.

When dealing with perceived barriers, the most frequently reported was identified in the lack of trust in “experimental” vaccines (37.8%), followed by the fear of neurological syndromes following the vaccine (36.9%), and more generally, the fear of side effects (36.0%), as well as those elicited by vaccine adjuvants (30.5%) and by heavy metals allegedly included in the vaccine formulate (30.2%). Similarly, 8.9% of respondents reported side effects after previous immunizations. Approximately 16.9% of participants reported a fear of autism, while 9.5% had a greater trust in alternative therapies, in preventive measures (7.7%), or in antimicrobial treatment (5.5%). Moreover, 5.5% of respondents aimed to reduce their number of injections. Interestingly, only 5.2% of participants were reportedly “against” vaccination practice and 3.4% identified meningococcus vaccines as scarcely affordable, while in 1.2% of cases, the vaccine was either not recommended by the competent general practitioner/pediatrician or not possible due to a previous condition of the respondents of their offspring.

### 3.5. Univariate Analysis

**Figure 4:**
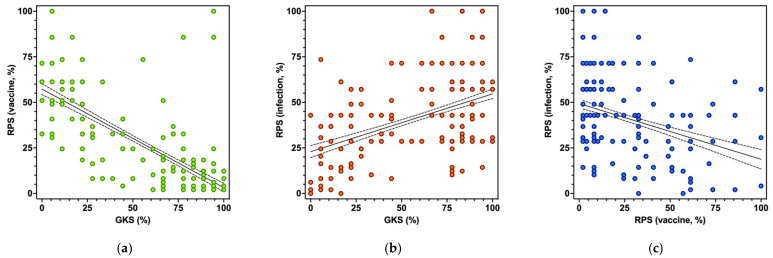
Correlation between General Knowledge Score (GKS), Risk Perception Score (RPS), on the side effects of Meningitis B vaccine and *Neisseria meningitidis* natural infection in participants with children (No. = 558, 55.2% of the original sample): subfigure (**a**) RPS on vaccines vs. GKS; subfigure (**b**) RPS on natural infection vs. GKS, subfigure (**c**) RPS on natural infection vs. RPS on vaccines. GKS was positively correlated with RPS on natural infection (Spearman’s rho = 0.432, *p* < 0.001) and negatively correlated with RPS on vaccine side effects (rho = −0.725, *p* < 0.001), while RPS on vaccines and natural infection were negatively correlated (rho = −0.238, *p* < 0.001).

**Figure A4:**
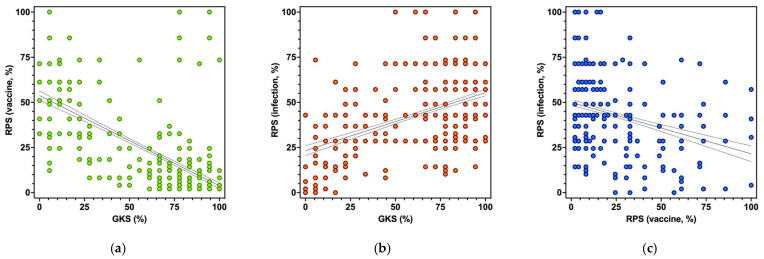
Correlation between General Knowledge Score (GKS), Risk Perception Score (RPS), on the side effects of Meningitis B vaccine and *Neisseria meningitidis* natural infection in all participants (No. = 1010). Subfigure (**a**) RPS on vaccines vs. GKS; subfigure (**b**) RPS on natural infection vs. GKS; subfigure (**c**), RPS on natural infection vs. RPS on vaccine. In fact, GKS was positively correlated with RPS on natural infection (Spearman’s rho = 0.400, *p* < 0.001) and negatively correlated with RPS on vaccine side effects (rho = −0.666, *p* < 0.001), while RPS on vaccines and natural infection were negatively correlated (rho = −0.235, *p* < 0.001).

As shown in Figure 4 (see also Table A5), GKS was positively correlated with RPS on natural infection (Spearman’s rho = 0.432, *p* < 0.001) and negatively correlated with RPS on vaccine side effects (rho = −0.725, *p* < 0.001), while RPS on vaccines and natural infection were negatively correlated (rho = −0.238, *p* < 0.001). Similar estimates for the sample as a whole are reported in Figure A4.

When cumulative scores were compared by vaccination status of the respondent, individuals who had been vaccinated against meningococcus serogroup B reported substantially higher estimates (74.7% ± 24.6 vs. 47.5% ± 34.5; W-W U 48,958.0, *p* < 0.001; see Figure 2) than those who had not, and similarly RPS on serogroup B meningococcus natural infections was higher among the vaccinated than the unvaccinated (50.9% ± 19.2 vs. 42.6% ± 21.1; M–W U 79,134.0, *p* < 0.001), while RPS was conversely lower among the vaccinated than the unvaccinated (12.8% ± 17.6 vs. 23.4% ± 23.6; M–W U 47,625.0, *p* < 0.001) (Figure 3a,b). Interestingly, substantial differences among respondents with and without children < 14 years old were reported for all cumulative scores (*p* < 0.001), with higher GKS and RPS for natural infections among individuals without offspring (57.6% ± 33.6 vs. 71.5% ± 23.1 and 46.5% ± 19.0 vs. 41.6% ± 22.3, respectively), while higher RPS on vaccine was scored by participants with any children aged less than 14 years than among those without offspring (26.2% ± 25.8 vs. 16.5% ± 17.8, respectively) (Table A6).

Table 5 provides the results of the univariate analysis for factors associated with having vaccinated offspring against *Neisseria meningitidis* serogroup B. As a total of 17 participants did not share the vaccine status of their offspring, estimates were calculated for 541 out of 558 potential respondents.

As shown in Table 5, having their offspring vaccinated against serogroup B meningococcus was positively associated with the male gender (29.6% vs. 16.0% among respondents having not reportedly vaccinated their offspring, *p* < 0.001), living in a municipality with more than 15,000 inhabitants (64.4% vs. 55.7%, *p* = 0.045), and reporting a better knowledge score (53.7% vs. 29.8%, *p* < 0.001), while it was negatively associated with reporting a higher RPS score (30.1% vs. 66.5%, *p* < 0.001). Nonetheless, offspring vaccination was more frequently reported among individuals reporting a positive attitude on vaccines, in general (88.9% vs. 51.1%, *p* < 0.001), as well as regarding serogroup B meningococcus (89.4% vs. 48.0%, *p* < 0.001); among subjects with higher confidence in vaccination services (81.5% vs. 44.3%, *p* < 0.001); and among respondents reporting higher confidence in vaccines, both in general (84.3% vs. 43.4%, *p* < 0.001), as well as regarding vaccines for meningococcus of serogroup B (89.4% vs. 47.7%, *p* < 0.001). Finally, vaccination of offspring was more frequently reported among participants having been vaccinated against meningococcus B (21.3% vs. 2.2% among those having not been vaccinated; *p* < 0.001) and meningococcus C (38.9% vs. 10.2%, *p* < 0.001), and having reportedly vaccinated their offspring against meningococcus C (81.7% vs. 37.8% among participants not having vaccinated their children against meningococcus of serogroup B; *p* < 0.001). Data on the sample as a whole are reported in Table A7.

### 3.6. Multivariable Analysis

Regression analysis (Table 6 and Table A8) included the following variables: male gender, living in a municipality greater than 15,000 inhabitants, higher GKS, higher RPS for vaccines, a favorable attitude towards vaccines (in general) and meningococcus vaccine, higher confidence in vaccination services, higher confidence in vaccines (in general) and meningococcus vaccine for serogroup B, having been vaccinated against meningococcus B and meningococcus C, and having previously vaccinated their offspring against meningococcus of serogroup C. Similar analyses for the sample as a whole are reported in Table A9.

Briefly, the male gender (aOR 3.184, 95%CI 1.772 to 5.721), living in a municipality >15,000 inhabitants (aOR 1.675, 95%CI 1.051 to 2.668), reporting a favorable attitude on meningococcus B vaccine (aOR 12.472, 95%CI 3.030 to 51.338), having been vaccinated against serogroup B (aOR 5.624, 95%CI 1.936 to 16.337) and/or serogroup C (aOR 2.652, 95%CI 1.442 to 4.872), and having previously vaccinated their offspring against serogroup C meningococcus (aOR 6.585, 95%CI 3.648 to 11.888) were characterized as positive effectors. On the contrary, having a higher risk perception on vaccines was identified as the only negative effector (aOR 0.429, 95%CI 0.241 to 0.765).

## 4. Discussion

To date, IMD cases in Italy are quite uncommon events. According to the national surveillance system for bacterial meningitis, a total of 26 cases of IMD were reported in 2021 in the whole of the Italian population (4 of them in Emilia Romagna; 15.4%), compared to 74 in 2020 (7 from Emilia Romagna; 9.4%), with a stark and sustained decline from the notification estimates of 2019 (190 cases, 8.9% from Emilia Romagna), but also for 2018 and 2017 (170 and 197 cases, respectively). The corresponding incidence rates ranged from 0.33 cases/100,000 population in 2017, 0.28 cases/100,000 population in 2018, and 0.31 cases/100,000 population in 2019, to eventually 0.12 cases/100,000 population in 2020 and 0.04 cases/100,000 population in 2021 [53]. Even though such figures are reasonably affected by substantial underreporting [19,28], Italian estimates were therefore quite lower than the European figures, ranging from an average of 0.6 cases/100,000 population for 2017 to 0.26 cases/100,000 population from 2020 (latest available data at the time of the survey) [23,54,55,56,57]. In other words, even though IMD is an important cause of morbidity, with a high lethality and high frequency of sequelae, often disabling, leading to high direct and indirect social costs, the actual cost-effectiveness rationale for general vaccination campaigns could be questioned [19,28,58]. Not, coincidentally, while the Italian National Vaccination Plan 2017–2019 and its further iteration 2023–2025 have stressed the need for reaching high coverage rates [19,25,26], MenB and MenC were eventually not included in the extended list of mandatory vaccines for individuals aged 0 to 16 years [27].

In other words, reaching and maintaining high vaccination rates for MenB and MenC still represent a main public health objective [19,23,28]. In this study, we specifically targeted KAP of parents of potential recipients of meningococcus B vaccination, as well as potential barriers and effectors of this preventive intervention. In fact, only 38.7% of participants had previously vaccinated their offspring against serogroup B meningococcus. Even though the comparison with official Italian data is complicated by the uneven immunization status of cohorts after 2004 in regard of MenB [38,39], this share is somehow consistent with estimates reported at the end of 2019 in Emilia Romagna (the Italian region comprising the analyzed provinces of Parma and Reggio Emilia): 83.1% at 24 months of age, 32.1% at 36 months of age, 29.1% at 48 months [38]. Eventually, the male gender, living in a municipality >15,000 inhabitants, reporting a favorable attitude on meningococcus B vaccine, having been vaccinated against serogroup B, and/or serogroup C, and having previously vaccinated their offspring against serogroup C meningococcus were characterized as positive effectors. Personal antecedents with meningococcal vaccines (both in the respondents and their offspring) are not only the most significant effectors but are highly consistent with the underlying health belief model (HBM) [29,59]. Even though it was originally developed in the 1950s, HBM is still widely applied in health behavior research [60,61,62]. The basic assumption of this model is that the beliefs about the susceptibility to a certain health threat, corresponding perceptions on the potential severity of that threat, and perceived benefits (and conversely, barriers) associated with a particular intervention will determine whether or not an individual will adopt that action [51,63]. Indeed, not only scoring a higher risk perception on vaccines was identified as the only negative effector—in other words, the higher the risk perception on vaccines, the lower are the reported vaccination rates among their offspring; however, when participants were asked to identify the most significant barriers towards vaccination, concerns on the potential side effects and a lack of trust in vaccines were characterized as the two most significant [50,64,65,66]. Interestingly, our results were not only consistent with the HBM, but also with more recent developments on the psychological antecedents of vaccinations, including confidence, constraints, complacency, and calculation [65]. As a matter of fact, when focusing on the main drivers and potential barriers reported by study participants, the majority of drivers were identified among factors associated with personal convenience (i.e., being protected against infectious diseases, avoiding meningitis and its complications) and the aim to protect individuals who cannot be vaccinated [65].

Indeed, our survey was somewhat consistent with previous studies performed on the determinants of meningococcus vaccination [67,68,69], and particularly so with research performed in Italy on both the MenB and MenC vaccines before the SARS-CoV-2 pandemic [70,71]. Compared to the study of Morrone et al. [71], our study had a very different strategy for the assessment of knowledge status, based on a previously validated questionnaire [40,41,42,43,50,52]. In our study, the overall knowledge status was far from optimal (57.6% ± 33.6) and some of the items specifically targeting meningococcal infection were particularly associated with lower performance in the knowledge test (Q1 to Q5). Still, we cannot rule out that some of the collected answers may have been affected by social desirability bias, with participants reporting higher rates of “common sense” or “socially appropriated and socially desirable” answers compared to their actual understanding of the assessed item of their will regarding offspring’s vaccination [40,72]. For instance, Q04 included a potentially ambiguous statement on meningococcal vaccines (i.e., “Vaccination against meningococcus reduces spread of bacteria, contributing to herd immunity and ultimately protecting those who cannot be vaccinated”). In fact, a key aspect of vaccination programs targeting meningococcal B serogroup is the limited effect of this immunization on the circulation of the pathogen, at least compared to the MenC and MenACXY vaccines. Indeed, in the late 1990s, the spread of the hypervirulent ST-11 clone of serogroup C meningococcus [4,13,73,74,75] led to the introduction of meningococcal C conjugate vaccine (MenC) in the national routine childhood immunization programs [13,15,73,74,76,77,78]. After that introduction, cases of IMD associated with serogroup C almost disappeared, not only among vaccinated individuals (reduction of 98%), but also among unvaccinated people (reduction of 90%), with the latter benefiting from the reduced mucosal carriage of this pathogen among vaccinated subjects [4,75]. On the contrary, a similar effect is not usually associated with MenB vaccine [17,18,19,20]. Therefore, Q04 may be used to infer social desirability bias, as most responders would not know this specific characteristic of MenB vaccine.

In comparison to other Italian reports, we specifically did not inquire whether respondents had any knowledge of MenB [71], but similarly to the report by de Waure et al. [29] on MenC, we assessed the vaccination status of offspring. In this regard, it is of some interest to stress how the share of respondents having reportedly vaccinated their children was strikingly different, i.e., 68.8% in the aforementioned study compared to 38.7% in our study (chi square 78.51, *p* < 0.001). In fact, not only is Italy usually acknowledged as geographically quite heterogenous in terms of health literacy [25,79,80], but also our sampling strategy could explain such heterogeneity. In both studies, sampled individuals included either people aged ≥18 years accessing the department of prevention of the local health authorities of Genoa, Savona, Roma, and Viterbo (two different regions, Liguria and Latium) [29] or parents of children from primary and middle schools from Naples and Salerno (region of Campania) [71]. As a consequence, our study not only reports on quite different areas (North-Western provinces of Emilia Romagna Region), but also on different individuals, either in terms of literacy, town size, or easiness to access the vaccination programs (which in Italy are region-based). 

### Limits 

Despite its potential interest, the present article is unfortunately affected by several limitations [81,82,83,84,85,86,87,88,89,90,91,92,93]. First of all, the present study had a cross-sectional design: by definition, cross-sectional studies have no dimension of time, so they cannot support conclusions on the risk factors of the targeted outcome, nor on causal relationships [83,84,85,86,87,88,89,90]. Without a well-defined dimension of time, cross-sectional studies can be poorly suited for examining conditions of short duration [92,93,94]: from this point of view, a cross-sectional design may be well suited for studying the acceptance of vaccine interventions [65,95], as the research would inquire about the overall status of the targeted individuals before the intervention [83,96,97], while retrieved information on infectious diseases and associated risk factors should be more cautiously assessed [94,95,98].

Second, our data are reasonably affected by some degree of self-selection bias, an issue substantially shared by all web-based studies [52,81,82]. Despite their reliability, proven cost-effectiveness, and turnaround times that are starkly reduced when compared to more conventional approaches, web-based surveys are affected by some sort of “self-selection” of participants, which ultimately leads to the over-sampling of certain sub-groups [83,84,85]. Having inquired about the very specific topic of MenB, the oversampling of subjects who were more familiar with the assessed topic is quite reasonable [81], as suggested by similarly designed cross-sectional studies [86,87,88]. Nonetheless, our sample over-represented subjects having high or even very high educational qualifications, as nearly two-thirds of them reported a university-level degree compared to 28% in the study of Morrone et al. [71], and nearly a quarter of them had some sort of background in healthcare settings. In other words, the estimates largely exceeded official figures from the Emilia Romagna Region for the University-level qualification (17.4% according to the Italian National Institute for Statistics, ISTAT; http://dati.istat.it/, accessed on 20 December 2022). A similar quantification of individuals with a documented background in healthcare settings, by 31 December 2018 (i.e., during the year before the present study), by the Regional Health Service of Emilia Romagna accounted for a total of 61,854 professionals (i.e., 1.4% of the total population) (https://salute.regione.emilia-romagna.it/trasparenza/personale-dipendente/dipendenti-siti-web-approfondimenti, accessed on 20 December 2022). As a consequence, we reasonably collected a substantial share of individuals who were particularly willing to contribute to our study because of a series of factors, including and not limited to their better literacy or younger age, an increased attitude to sharing personal information through Internet access, greater knowledge and/or interest about the assessed topic, and a stronger will to contribute to health-care-related research because of their healthcare background [81]. Not coincidentally, we clearly oversampled individuals of the female gender (78.1%), a feature that was still shared by previous Italian studies on meningococcal vaccines [29,71], and that is often reported in KAP surveys on healthcare topics [59,89,90,91,92,93].

Similarly, as the questionnaire was not externally validated, we cannot rule out some degree of declarative bias. In other words, some of the respondents could not truly adhere to our inclusion criteria, particularly when dealing with the geographical recruitment of study participants. Moreover, in analogy with the items of the knowledge test, some participants may have reported “socially desirable” attitudes instead of their actual ones, inflating the actual acceptance of vaccines and preventive interventions [40,72]. In this regard, the lack of external validation and the limited set of information we were able to retrieve in order to cope with the privacy and confidentiality requirements may have led to the uneven sampling of participating parents. More precisely, it is quite likely that some of the respondents were parents of the very same index child, eventually over-representing their corresponding practices over those of “single parents”.

Third, even though our sample fulfilled the sample size requirements, it may be acknowledged as relatively small compared to the whole of the Italian population, particularly when stressing the distinctive Italian regional patterns of healthcare settings. On the one hand, our sample was substantially in line with estimates from other Italian studies, and its geographical settings may contribute to the overall understanding of the heterogenous acceptance of this vaccination in Italy shortly before the inception of the SARS-CoV-2 pandemic [29,71]. 

In this regard, the very same timeframe of our inquiry may represent both a substantial limit and also a potential strength of the present study, as the provinces of Parma and Reggio Emilia were extensively affected by the SARS-CoV-2 pandemic [99,100,101], particularly between March and May 2020; as a consequence, our study could share a “historical” picture of KAP on vaccination policies on MenB shortly before an event that has reasonably affected the overall acceptance of vaccines, potentially contributing to future studies as a reference [31,38,39,83,85]. 

Last but not least, we cannot rule out that our results may have been influenced by background factors, such as the coverage by conventional and social media of meningococcal infections. An extensive representation of certain topics in the media may reasonably lead to overstating the true significance of this pathogen from a public health point of view [102]. While the appropriate measurement of the qualitative and quantitative coverage of a specific topic by conventional media is particularly difficult to achieve, Google Trends™ may represent a reliable proxy for new media coverage [103,104,105,106]. Google Trends^TM^ is an open online tool [107,108,109], where web searches on a certain topic are reported as relative search volumes (RSV), i.e., the normalized value ranging from 0 to 100 and proportional to the ratio between the keyword-related queries and the total number of web queries. The assessment of a potential correlation existed between GKS and RPS on the one hand, and RSV on meningitis from 1 July to 31 December 2019 (i.e., the exact timeframe when the web survey was made available) on the other hand. Eventually, during the study period, a substantial correlation with RSV on meningitis was found for both components of RPS, with a negative correlation for RPS on natural infection (Spearman’s rho = −0.198; *p* < 0.001) and a positive correlation with RPS on vaccines (Spearman’s rho = 0.272; *p* < 0.001) [108,109]. In other words, the risk perception on MenB among study participants was seemingly influenced by new media coverage, urging for the cautious interpretation of our results. Our estimates should therefore be acknowledged as strictly dependent on the exact timeframe of this study, and we cannot rule out that a follow-up study would lead to a different outcome.

## 5. Conclusions

Despite some substantial shortcomings associated with its design and timeframe, our study suggests that in the few months before the inception of the SARS-CoV-2 pandemic, the acceptance of MenB vaccination among parents of children aged less than 14 years in the provinces of Parma and Reggio Emilia was far from optimal. In fact, a positive attitude towards vaccines and vaccinations was identified as the main effector of MenB acceptance, while the usual effectors of vaccine hesitancy/propensity seemingly had a more limited role. Interventions on the general population aimed to improve confidence (i.e., the trust in vaccines), compliance (i.e., the support for people opting to be vaccinated through improved access to vaccination services), and acknowledgment of the collective responsibility associated with vaccine interventions, as well as preventing actual constraints and the sharing of false beliefs on infectious diseases and their preventive measures, could therefore increase vaccination acceptance in both targeted individuals and their offspring.

## Figures and Tables

**Table 1:** Demographics of the 1010 subjects participating in the survey on the acceptance of Neisseria meningitis B serogroup (MenB) vaccine (provinces of Parma and Reggio Emilia, 2019) by their parental status (i.e., having or not having any children aged less than 14 years).

Variable	Only Participants with Children <14 Years No./558, %	Participants without Children <14 Years No./452, %	Chi Squared Test *p* Value
Gender			<0.001
Male	122, 21.9%	146, 32.3%	
Female	436, 78.1%	306, 67.7%	
Age			0.181
<40 years	295, 52.9%	258, 57.1%	
≥40 years	263, 47.1%	194, 42.9%	
Literacy			0.030
Primary School	20, 3.6%	15, 3.3%	
Secondary School	174, 31.2%	177, 39.2%	
University or higher	364, 65.2%	260, 57.5%	
Residency			<0.001
Municipality <5000 inhabitants	64, 11.5%	86, 19.0%	
Municipality 5000–15,000 inhabitants	161, 28.9%	74, 16.4%	
Municipality >15,000 inhabitants (1)	176, 31.5%	148, 32.7%	
Main Municipality (2)	157, 28.1%	137, 30.3%	
n.a.	0, -	7, 1.5%	
Size of Household			<0.001
Single	0, -	40, 8.8%	
2 persons	12, 2.2%	162, 35.8%	
3 persons	230, 41.2%	100, 22.1%	
4 persons	252, 45.2%	106, 23.5%	
5 persons or more	60, 10.8%	37, 8.2%	
n.a.	4, 0.7%	7, 1.5%	
Household including subjects ≥ 65 y.o.	74, 13.3%	94, 20.8%	0.001
Background in healthcare settings	149, 26.7%	161, 35.6%	0.002

Notes: (1) with the exclusion of main municipalities of Parma and Reggio Emilia; (2) Parma (196,330 inhabitants) and Reggio Emilia (169,640 inhabitants).

**Table 2:** Items included in the knowledge test performed on 558 subjects from the provinces of Parma and Reggio Emilia participating in the present survey (2019). Internal consistency was calculated by means of Cronbach’s alpha (0.938).

Statement	Correct Answer	No./558, %
Q01. Meningococcus causes around 40–50% of all bacterial meningitis in children aged 2 years or more, and over 70% in children aged 5 years or more. Overall, it causes half of all bacterial meningitis in all infants.	TRUE	237, 42.5%
Q02. The mortality of meningococcal meningitis is around 30%, irrespective of therapy.	TRUE	315, 56.5%
Q03. Early symptoms of meningococcal meningitis are specific, allowing a prompt and appropriate treatment by healthcare providers.	FALSE	373, 66.8%
Q04. Vaccination against meningococcus reduces spread of bacteria, contributing to herd immunity and ultimately protecting those who cannot be vaccinated.	TRUE	368, 65.9%
Q05. MenB vaccine may be associated with other vaccinations and vaccine formulates, not increasing the number of accesses to vaccination services.	TRUE	227, 40.7%
Q06. Vaccine additives are not harmful to human beings.	TRUE	271, 48.6%
Q07. Vaccines against measles may cause severe damages to the central nervous system.	FALSE	313, 56.1%
Q08. Influenza vaccine may cause severe side effects, potentially lethal.	FALSE	210, 37.6%
Q09. Measles vaccine may elicit autism.	FALSE	381, 68.3%
Q10. Some vaccinations may cause diabetes.	FALSE	311, 55.7%
Q11. Some autoimmune diseases may be elicited by vaccines.	FALSE	252, 45.2%
Q12. Vaccines are useless, as infectious diseases may be always treated with specific therapies.	FALSE	463, 83.0%
Q13. Vaccination shots increase probabilities of allergic reactions.	FALSE	304, 54.5%
Q14. Without vaccinations, smallpox would still exist.	TRUE	398, 71.3%
Q15. The efficacy of vaccines and vaccination programs has been repetitively proved.	TRUE	379, 67.9%
Q16. Children would be more resistant to infectious disease without vaccinations.	FALSE	349, 62.5%
Q17. Many immunizations are performed too early: as a consequence, the immune system of children is not allowed to fully develop by itself.	FALSE	315, 56.5%
Q18. The immune system may be compromised by receiving too many vaccines in pediatric age.	FALSE	323, 57.9%

**Table 3:** Items associated with risk perception and attitudes on invasive meningococcal diseases and meningococcal vaccines of 558 subjects participating in the survey on the acceptance of Neisseria meningitis B serogroup (MenB) vaccine (provinces of Parma and Reggio Emilia, 2019).

Variable	Only Participants with Children <14 Years No./558, %
RPS^inf^ > median	210, 37.6%
RPS^vac^ > median	288, 51.6%
Perception on *Neisseria meningitidis*	
Severe/Very Severe	496, 88.9%
Frequent/Highly Frequent	104, 18.6%
Perception on Neisseria Vaccines’ side effects	
Severe/Very Severe	159, 28.5%
Frequent/Highly Frequent	147, 26.3%
Attitude Towards Vaccines: somehow favorable	
Vaccines, in general	367, 65.8%
Meningitis vaccines	354, 63.4%
Previously vaccinated against MenC	120, 21.5%
Previously vaccinated against MenB	53, 9.5%
Offspring vaccinated against MenB	216, 38.7%
Confidence in Vaccination Services (high/very high)	326, 58.4%
Confidence in Vaccines (high/very high)	329, 59.0%
Confidence in MenB (high/very high)	358, 64.2%

Note: RPS^inf^ = Risk Perception Score, meningococcal natural infection; RPS^vac^ = Risk Perception Score, side effects of vaccines.

**Table 4:** Drivers and barriers on *Neisseria meningitidis* B vaccine as reported by study participants. Estimates are reported on participants with offspring and who shared the vaccination status of their children (i.e., 541 out of 558 potential participants, 97.0%).

Drivers	Child Vaccinated (No./216)
It is included in the National Vaccination Plan	77, 35.6%
Suggested by general practitioner/pediatrician	44, 20.4%
Meningitidis is a severe disease	139, 64.4%
In order to be protected against as many infectious diseases as possible	157, 72.7%
In order to be protected against meningitis	121, 56.0%
An acquaintance has been affected by meningitis	16, 7.4%
In order to avoid bacterial meningitis	152, 70.4%
In order to avoid complications of meningitis	169, 78.2%
In order to avoid transmission of meningitis	116, 53.7%
In order to protect subjects who cannot be vaccinated	134, 62.2%
**Barriers**	**Child not Vaccinated** **(No./325, %)**
I am against vaccines (in general)	17, 5.2%
Preference in other preventive measures	25, 7.7%
Greater trust in antimicrobial treatment	18, 5.5%
Greater trust in alternative therapies (e.g., homeopathy)	31, 9.5%
Cannot be vaccinated	4, 1.2%
Side effects after previous vaccination	29, 8.9%
Fear of side effects	117, 36.0%
Lack of trust in “experimental” vaccines	123, 37.8%
Fear of neurological disorders induced by vaccines (e.g., Guillaume Barré syndrome, multiple sclerosis)	120, 36.9%
Fear of developing autism after vaccine	55, 16.9%
Fear of side effects elicited by vaccine adjuvants	99, 30.5%
Fear of side effects elicited by heavy metals included in the vaccine	98, 30.2%
Not recommended by general practitioner/pediatrician	4, 1.2%
Aiming to reduce the number of injections	18, 5.5%
Vaccine not affordable	11, 3.4%

**Table 5:** Univariate analysis for factors associated with having vaccinated offspring against *Neisseria meningitidis* serogroup B (MenB). A total of 17 participants reporting any child in the demographics were removed from the analysis, as they did not share the vaccine status of their offspring (final sample, 541 out of 558 potential respondents, 97.0%). Calculations were performed by means of chi squared test.

	Vaccinated Children		Chi Squared Test *p* Value
	Yes No./216, %	No No./325, %	
Male gender	64, 29.6%	52, 16.0%	<0.001
Age ≥40 years	94, 43.5%	156, 48.0%	0.306
Literacy: University or higher	149, 69.0%	206, 63.1%	0.157
Municipality >15,000 inhabitants	139, 64.4%	181, 55.7%	0.045
Migration Background	4, 1.9%	14, 4.3%	0.119
Household Characteristics			
… including ≥3 people	208, 96.3%	317, 97.5%	0.404
… including subjects ≥65 y.o.	32, 14.8%	39, 12.0%	0.342
Background in Healthcare Settings	66, 30.6%	83, 25.5%	0.201
General Knowledge Score > median	116, 53.7%	97, 29.8%	<0.001
Risk Perception Score > median			
… of Natural infection	91, 42.1%	116, 35.7%	0.131
… of Vaccines	65, 30.1%	216, 66.5%	<0.001
Attitude Towards Vaccines: somehow favorable			
Vaccines, in general	192, 88.9%	166, 51.1%	<0.001
Meningitis vaccines	193, 89.4%	156, 48.0%	<0.001
Confidence in Vaccination Services (high/very high)	176, 81.5%	144, 44.3%	<0.001
Confidence in Vaccines (high/very high)	182, 84.3%	141, 43.4%	<0.001
Confidence in MenB Vaccines (high/very high)	193, 89.4%	155, 47.7%	<0.001
Previously vaccinated against MenB	46, 21.3%	7, 2.2%	<0.001
Previously vaccinated against MenC	84, 38.9%	33, 10.2%	<0.001
Vaccinated children against MenC	174, 81.7%	123, 37.8%	<0.001

**Table 6:** Multivariable analysis of factors associated with the outcome variable of having vaccinated offspring against *Neisseria meningitidis* serogroup B (MenB). The analyses only included individuals with offspring, and who also shared their offspring’s vaccination status (No. = 541 out of 558 potential respondents, 97.0%). Adjusted Odds Ratios (aOR) were calculated by means of a binary logistic regression analysis that included as effector variables all factors that in univariate analysis were associated with the outcome variable with *p* < 0.05.

Variable	aOR	95% Confidence Interval	
Male gender	3.184	1.772	5.721
Municipality >15,000 inhabitants	1.675	1.051	2.668
General Knowledge Score > median	0.644	0.365	1.135
Risk Perception Score for Vaccines > median	0.429	0.241	0.765
Attitude Towards Vaccines: somehow favorable			
Vaccines, in general	0.585	0.140	2.441
Meningitis vaccines	12.472	3.030	51.338
Confidence in Vaccination Services (high/very high)	0.932	0.386	2.254
Confidence in Vaccines (high/very high)	1.629	0.626	4.238
Confidence in MenB Vaccines (high/very high)	0.204	0.040	1.028
Previously vaccinated against MenB	5.624	1.936	16.337
Previously vaccinated against MenC	2.652	1.442	4.874
Previously vaccinated children against MenC	6.585	3.648	11.888

**Figure A1:**
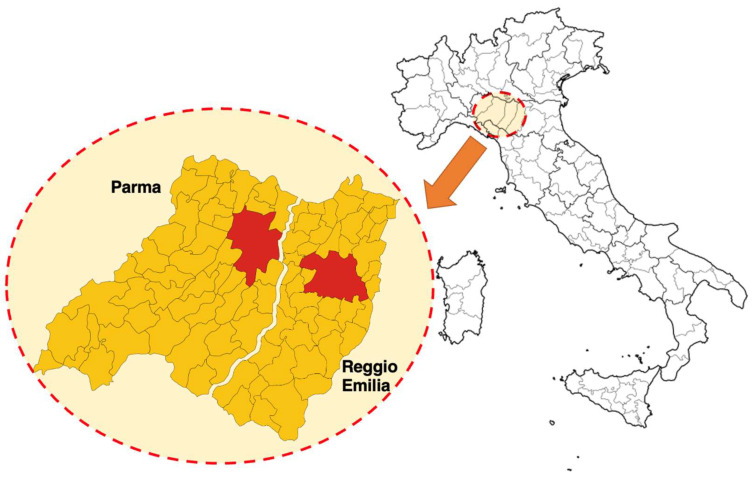
Geographical areas involved in the survey (i.e., provinces of Parma and Reggio Emilia). Original maps available from open source material: https://it.m.wikipedia.org/wiki/File:Map_of_comune_of_Reggio_nell%27Emilia_%28province_of_Reggio_Emilia,_region_Emilia-Romagna,_Italy%29.svg, https://it.m.wikipedia.org/wiki/File:Map_of_comune_of_Parma_(province_of_Parma,_region_Emilia-Romagna,_Italy).svg and https://it.m.wikipedia.org/wiki/File:Italy_map_with_provinces.svg (accessed on 16 January 2023).

## Data Availability

Data are available on request.

## Supplementary Materials

The following supporting information can be downloaded at: https://www.mdpi.com/article/10.3390/vaccines11030508/s1, File S1: STROBE checklist; File S2: Authors’ translation of the informed consent; File S3: Authors’ translation of the questionnaire; File S4: Authors’ translation of the Gazzetta Ufficiale No. 76, dated 31 March 2008.

## Appendix A

**Table A1:** Characteristics of 1010 subjects participating in the survey on the acceptance of Neisseria meningitis B serogroup (MenB) vaccine (provinces of Parma and Reggio Emilia, 2019).

Variable	All participants No./1010, %
Gender	
Male	268, 26.5%
Female	742, 73.5%
Age	
<40 years	553, 54.8%
≥40 years	457, 45.2%
Literacy	
Primary School	35, 3.5%
Secondary School	351, 34.8%
University or higher	524, 61.8%
Residency	
Municipality <5000 inhabitants	150, 14.9%
Municipality 5000–15,000 inhabitants	235, 23.3%
Municipality >15,000 inhabitants	324, 32.1%
Main Municipality	294, 29.1%
n.a.	7, 0.7%
Size of Household	
Single	40, 4.0%
2 persons	174, 17.2%
3 persons	330, 32.7%
4 persons	358, 35.4%
5 persons or more	97, 9.6%
Household including subjects <14 y.o.	558, 55.2%
Household including subjects ≥65 y.o.	168, 16.6%
Background in Healthcare Settings	310, 30.7%
General Knowledge Score > median	400, 39.6%
Confidence in Vaccination Services (high/very high)	724, 71.7%
Confidence in Vaccines (high/very high)	719, 71.2%
Confidence in MenB (high/very high)	751, 74.4%
Risk Perception Score (Infection) > median	441, 43.7%
Risk Perception Score (Vaccine) > median	464, 45.9%
Perception on *Neisseria meningitidis*	
Severe/Very Severe	927, 91.8%
Frequent/Highly Frequent	196, 19.4%
Perception on Neisseria Vaccines’ side effects	
Severe/Very Severe	214, 21.2%
Frequent/Highly Frequent	184, 18.2%
Attitude Towards Vaccines: somehow favorable	
Vaccines, in general	780, 77.2%
Meningitis vaccines	753, 74.6%
Previously vaccinated against MenC	285, 28.2%
Previously vaccinated against MenB	150, 14.9%

**Table A2:** Normality tests of summary scores. K2 = D’Agostino–Pearson’s K2, GKS = General Knowledge Score, RPS = Risk Perception Score.

	GKS (K2, *p* Value)	RPS (Vaccines) (K2, *p* Value)	RPS (Infection) (K2, *p* Value)
All participants	149.5, *p* < 0.001	219.7, *p* < 0.001	6.830, *p* = 0.033
Only participants with children	8486.0, *p* < 0.001	72.75, *p* < 0.001	11.36, *p* = 0.003

**Table A3:** Items included in the knowledge test performed on 1010 subjects from the provinces of Parma and Reggio Emilia participating in the present survey (2019). Internal consistency was calculated by means of Cronbach’s alpha (0.938).

Statement	Correct Answer	All Participants No./1010, %	Only Participants with Children <14 Years No./558, %	Participants without Children <14 Years No./452, %
Q01. Meningococcus causes around 40–50% of all bacterial meningitis in children aged 2 years or more, and over 70% in children aged 5 years or more. Overall, it causes half of all bacterial meningitis in all infants.	TRUE **	471, 46.6%	237, 42.5%	234, 51.8%
Q02. The mortality of meningococcal meningitis is around 30%, irrespective of therapy.	TRUE	591, 58.5%	315, 56.5%	276, 61.1%
Q03. Early symptoms of meningococcal meningitis are specific, allowing a prompt and appropriate treatment by healthcare providers.	FALSE **	632, 62.6%	373, 66.8%	259, 57.3%
Q04. Vaccination against meningococcus reduces spread of bacteria, contributing to herd immunity and ultimately protecting those who cannot be vaccinated.	TRUE ***	756, 74.9%	368, 65.9%	388, 85.8%
Q05. MenB vaccine may be associated with other vaccinations and vaccine formulates, not increasing the number of accesses to vaccination services.	TRUE ***	480, 47.5%	227, 40.7%	253, 56.0%
Q06. Vaccine additives are not harmful to human beings.	TRUE **	535, 53.0%	271, 48.6%	264, 58.4%
Q07. Vaccines against measles may cause severe damages to the central nervous system.	FALSE ***	669, 66.2%	313, 56.1%	356, 78.8%
Q08. Influenza vaccine may cause severe side effects, potentially lethal.	FALSE ***	433, 42.9%	210, 37.6%	223, 49.3%
Q09. Measles vaccine may elicit autism.	FALSE ***	770, 76.2%	381, 68.3%	389, 86.1%
Q10. Some vaccinations may cause diabetes.	FALSE *	595, 58.9%	311, 55.7%	284, 62.8%
Q11. Some autoimmune diseases may be elicited by vaccines.	FALSE ***	550, 54.5%	252, 45.2%	298, 65.9%
Q12. Vaccines are useless, as infectious diseases may be always treated with specific therapies.	FALSE ***	895, 88.6%	463, 83.0%	432, 95.6%
Q13. Vaccination shots increase probabilities of allergic reactions.	FALSE *	580, 57.4%	304, 54.5%	276, 61.1%
Q14. Without vaccinations, smallpox would still exist.	TRUE ***	817, 80.9%	398, 71.3%	419, 92.7%
Q15. The efficacy of vaccines and vaccination programs has been repetitively proved.	TRUE ***	800, 79.2%	379, 67.9%	421, 93.1%
Q16. Children would be more resistant to infectious disease without vaccinations.	FALSE ***	736, 72.9%	349, 62.5%	387, 85.6%
Q17. Many immunizations are performed too early: as a consequence, the immune system of children is not allowed to fully develop by itself.	FALSE **	612, 60.6%	315, 56.5%	297, 65.7%
Q18. The immune system may be compromised by receiving too many vaccines in pediatric age.	FALSE ***	684, 67.7%	323, 57.9%	361, 79.9%

Note: * = chi squared test, difference between participants with and without child aged <14 years at *p* of 0.01 to 0.05; ** = chi squared test, difference between participants with and without child aged <14 years at *p* of 0.001 to 0.01; *** = chi squared test, difference between participants with and without child aged <14 years at *p* < 0.001.

**Table A4:** Drivers and barriers on *Neisseria meningitidis* B vaccine as reported by all study participants (No. = 1010).

Drivers (Vaccinated)	All Participants (No./150, %)	Only Participants with Children <14 Years (No./53, %)	Participants without Children <14 Years (No./97, %)
It is included in the National Vaccination Plan **	91, 60.7%	24, 45.3%	67, 69.1%
Suggested by general practitioner	32, 21.3%	12, 22.6%	20, 20.6%
Meningitidis is a severe disease	119, 79.3%	38, 71.7%	81, 83.5%
In order to be protected against as many infectious diseases as possible ***	115, 76.7%	31, 58.5%	84, 86.6%
In order to be protected against meningitis	90, 60.0%	33, 62.3%	57, 58.8%
A friend has been affected by meningitis	22, 14.7%	8, 15.1%	14, 14.4%
In order to avoid bacterial meningitis	110, 73.3%	42, 79.2%	68, 70.1%
In order to avoid complications of meningitis	117, 78.0%	39, 73.6%	78, 80.4%
In order to avoid transmission of meningitis	94, 62.7%	30, 56.6%	64, 66.0%
In order to protect subjects who cannot be vaccinated	110, 73.3%	41, 77.4%	69, 71.1%
**Barriers** **(Not vaccinated)**	**All Participants** **(No./860, %)**	**Only Participants with Children < 14 Years** **(No./505, %)**	**Participants without Children < 14 Years** **(No./355, %)**
I am against vaccines (in general) *	21, 2.4%	17, 3.4%	4, 1.1%
Preference in other preventive measures	63, 7.3%	33, 6.5%	30, 8.5%
Greater trust in antimicrobial treatment ***	21, 2.4%	21, 4.2%	0, -
Greater trust in alternative therapies (e.g., homeopathy) ***	35, 4.1%	35, 6.9%	0, -
Cannot be vaccinated	30, 3.5% *	11, 2.2%	19, 5.4%
Side effects after previous vaccination ***	47, 5.5%	44, 8.7%	3, 0.8%
Fear of side effects ***	152, 17.7%	140, 27.7%	12, 3.4%
Lack of trust in “experimental” vaccines ***	173, 20.1%	146, 28.9%	27, 7.6%
Fear of neurological disorders induced by vaccines (e.g., Guillaume Barré syndrome, multiple sclerosis) ***	155, 18.0%	143, 28.3%	12, 3.4%
Fear of developing autism after vaccine ***	85, 9.9%	70, 13.9%	15, 4.2%
Fear of side effects elicited by vaccine adjuvants ***	140, 16.3%	122, 24.2%	18, 5.1%
Fear of side effects elicited by heavy metals included in the vaccine ***	146, 17.0%	125, 24.8%	21, 5.9%
Not recommended by general practitioner ***	69, 8.0%	8, 1.6%	61, 17.2%
Aiming to reduce the number of injections	41, 4.8%	25, 5.0%	16, 4.5%
Vaccine not affordable	60, 7.0% ***	13, 2.6%	47, 13.2%

Note: * = chi squared test, difference between participants with and without child aged <14 years at *p* of 0.01 to 0.05; ** = chi squared test, difference between participants with and without child aged <14 years at *p* of 0.001 to 0.01; *** = chi squared test, difference between participants with and without child aged <14 years at *p* < 0.001.

**Table A5:** Correlation between General Knowledge Score (GKS), Risk Perception Score (RPS) for *Neisseria meningitidis* serogroup B vaccine (MenB) and natural infection (rho = Spearman’s rank correlation coefficient).

	GKS	RPS (Vaccines)	RPS (Infection)
	rho, *p* Value	rho, *p* Value	rho, *p* Value
All participants (No. = 1010, 100%)			
GKS	-	−0.666, *p* < 0.001	0.400, *p* < 0.001
RPS (Vaccines)	−0.666, *p* < 0.001	-	−0.235, *p* < 0.001
RPS (Infection)	0.400, *p* < 0.001	−0.235, *p* < 0.001	-
Only participants with children (No. = 558, 55.2%)			
GKS	-	−0.725, *p* < 0.001	0.432, *p* < 0.001
RPS (Vaccines)	−0.725, *p* < 0.001	-	−0.238, *p* < 0.001
RPS (Infection)	0.432, *p* < 0.001	−0.238, *p* < 0.001	-

**Table A6:** Comparison between General Knowledge Score (GKS), Risk Perception Score (RPS) for *Neisseria meningitidis* serogroup B vaccine (MenB) and natural infection. Note: M–W = Mann–Whitney.

	GKS		RPS (Vaccines)		RPS (Infection)	
	Average ± SD	M–W U, *p* Value	Average ± SD	M–W U, *p* Value	Average ± SD	M–W U, *p* Value
All participants (No. = 1010, 100%)						
Vaccinated against MenB	77.6 ± 21.5	84,557.5	12.8 ± 17.6	47,625.0	50.9 ± 19.2	79,134.0
Not vaccinated	61.4 ± 30.8	*p* < 0.001	23.4 ± 23.6	*p* < 0.001	42.6 ± 21.1	*p* < 0.001
Only participants with children (No. = 558, 55.2%)						
Children vaccinated for MenB	74.7 ± 24.6	48,958.0	16.0 ± 21.3	19,615.0	44.8 ± 20.5	39,307.0
Not vaccinated	47.5 ± 34.5	*p* < 0.001	32.9 ± 26.2	*p* < 0.001	39.8 ± 23.7	*p* = 0.017
All participants (No. = 1010, 100%)						
Participants with children < 14 years old	57.6 ± 33.6	104,821.5	26.2 ± 25.8	148,971.0	46.5 ± 19.0	108,074.5
Participants without children < 14 years old	71.5 ± 23.1	*p* < 0.001	16.5 ± 17.9	*p* < 0.001	41.6 ± 22.3	*p* < 0.001

**Table A7:** Univariate analysis for factors associated with having been vaccinated against *Neisseria meningitidis* serogroup B (MenB). Calculations were performed on the sample as a whole (No. = 1010) by means of a chi squared test.

	Vaccinated against MenB		Chi Squared Test *p* Value
	Yes No./150, %	No No./860, %	
Male gender	58, 38.7%	210, 24.4%	<0.001
Age ≥40 years	35, 23.3%	422, 49.1%	<0.001
Literacy: University or higher	71, 47.3%	553, 64.3%	<0.001
Municipality >15,000 inh.	104, 69.3%	514, 59.8%	0.027
Migration Background	0, -	21, 2.4%	0.053
Household Characteristics			
… including 3 or more people	121, 80.7%	664, 77.2%	0.348
… including subjects <14 y.o.	53, 35.3%	505, 58.7%	<0.001
… including subjects ≥65 y.o.	40, 26.7%	128, 14.9%	<0.001
Background in Healthcare Settings	47, 31.3%	263, 30.6%	0.854
General Knowledge Score > median	76, 50.7%	324, 37.7%	0.003
Risk Perception Score > median			
Natural infection	79, 52.7%	362, 42.1%	0.016
Vaccines	40, 26.7%	424, 49.3%	<0.001
Attitude Towards Vaccines: somehow favorable			
Vaccines, in general	142, 94.7%	638, 74.2%	<0.001
Meningitis vaccines	143, 95.3%	610, 70.9%	<0.001
Confidence in Vaccination Services (high/very high)	143, 95.3%	581, 67.6%	<0.001
Confidence in Vaccines (high/very high)	135, 90.0%	584, 67.9%	<0.001
Confidence in MenB Vaccines (high/very high)	143, 95.3%	608, 70.7%	<0.001
Previously vaccinated against MenC	124, 82.7%	161, 18.7%	<0.001

**Table A8:** Multivariable analysis of factors associated with the outcome variable of having vaccinated offspring against *Neisseria meningitidis* serogroup B (MenB). The analyses only included individuals with offspring, and who also shared their offspring’s vaccination status (No. = 541 out of 558 potential respondents, 97.0%). Analyses were performed by means of a binary logistic regression analysis that included as effector variables all factors that in univariate analysis were associated with the outcome variable with *p* < 0.05. Notes: B = predicted change in Log Odds—for 1-unit change in predictor; S.E. = standard error of B.

Variable	B	S.E.	Wald Coefficient	*p* Value
Male gender	1.158	0.299	15.002	<0.001
Municipality >15,000 inhabitants	0.516	0.238	4.708	0.030
General Knowledge Score > median	−0.440	0.290	2.314	0.128
Risk Perception Score for Vaccines > median	−0.846	0.295	8.233	0.004
Attitude Towards Vaccines: somehow favorable				
Vaccines, in general	−0.536	0.729	0.541	0.462
Meningitis vaccines	2.523	0.722	12.219	<0.001
Confidence in Vaccination Services (high/very high)	−0.070	0.450	0.024	0.877
Confidence in Vaccines (high/very high)	0.488	0.488	0.999	0.318
Confidence in MenB Vaccines (high/very high)	−1.591	0.826	3.712	0.054
Previously vaccinated against MenB	1.727	0.544	10.077	0.002
Previously vaccinated against MenC	0.975	0.311	9.854	0.002
Previously vaccinated children against MenC	1.885	0.301	39.113	<0.001
Constant	−2.466	0.434	32.273	<0.001

**Table A9:** Multivariable analysis of factors associated with the outcome variable of having been vaccinated against *Neisseria meningitidis* serogroup B (MenB). Adjusted Odds Ratios (aOR) were calculated by means of a binary logistic regression analysis that included as effector variables all factors that in univariate analysis were associated with the outcome variable with *p* < 0.05.

Variable	aOR	95% Confidence Interval	
Male gender	1.447	0.908	2.308
Age ≥40 years	0.514	0.312	0.847
Literacy: University or higher	0.657	0.413	1.045
Municipality >15,000 inh.	1.350	0.816	2.235
Household Characteristics			
… including subjects <14 y.o.	0.672	0.425	1.063
… including subjects ≥65 y.o.	1.107	0.645	1.899
General Knowledge Score > median	0.759	0.472	1.222
Risk Perception Score > median			
Natural infection	1.043	0.660	1.649
Vaccines	0.977	0.548	1.742
Attitude Towards Vaccines: somehow favorable			
Vaccines, in general	0.325	0.073	1.460
Meningitis vaccines	5.355	1.565	18.328
Confidence in Vaccination Services (high/very high)	10.325	2.783	38.308
Confidence in Vaccines, in general (high/very high)	0.365	0.131	1.015
Confidence in MenB Vaccine (high/very high)	0.643	0.168	2.458
Previously vaccinated against MenC	14.978	8.983	24.974
