# Supplementary material for: Invasive Meningococcal Disease and Meningococcal Serogroup B Vaccination in Adults and Their Offspring: Knowledge, Attitudes, and Practices in Italy (2019)
Source: Vaccines (Basel). 2023 Feb 22;11(3):508. doi: 10.3390/vaccines11030508 (PMC10058645; doi:10.3390/vaccines11030508)
Supplement: Supplementary file 1 [file vaccines-11-00508-s001.zip › SUPPLEMENTARY FILES/MEN - FILE S4.pdf]

SERIE GENERALE

Spediz. abb. post. 45% - art. 2, comma 20/b
Legge 23-12-1996, n. 662 - Filiale di Roma

Anno 149° — Numero 76

GAZZETTA UFFICIALE

DELLA REPUBBLICA ITALIANA

PARTE PRIMA **Roma - Lunedì, 31 marzo 2008** SI PUBBLICA TUTTI I GIORNI NON FESTIVI

DIREZIONE E REDAZIONE PRESSO IL MINISTERO DELLA GIUSTIZIA - UFFICIO PUBBLICAZIONE LEGGI E DECRETI - VIA ARENULA 70 - 00186 ROMA
AMMINISTRAZIONE PRESSO L'ISTITUTO POLIGRAFICO E ZECCA DELLO STATO - LIBRERIA DELLO STATO - PIAZZA G. VERDI 10 - 00198 ROMA - CENTRALINO 06 85081

La **Gazzetta Ufficiale,** *oltre alla* **Serie generale,** *pubblica cinque Serie speciali, ciascuna contraddistinta da autonoma numerazione:*

1ª Serie speciale: *Corte costituzionale* (pubblicata il mercoledì)
2ª Serie speciale: *Comunità europee* (pubblicata il lunedì e il giovedì)
3ª Serie speciale: *Regioni* (pubblicata il sabato)
4ª Serie speciale: *Concorsi ed esami* (pubblicata il martedì e il venerdì)
5ª Serie speciale: *Contratti pubblici* (pubblicata il lunedì, il mercoledì e il venerdì)

SOMMARIO

DECRETI, DELIBERE E ORDINANZE MINISTERIALI

Presidenza del Consiglio dei Ministri
DIPARTIMENTO PER L'INNOVAZIONE E LE TECNOLOGIE

DECRETO 3 agosto 2007.

Rifinalizzazione delle risorse al progetto «La P.A. che si vede» . . . . Pag. 5

Ministero degli affari esteri

DECRETO 15 febbraio 2008.

Disciplina delle articolazioni interne degli uffici di livello dirigenziale generale istituiti presso l'amministrazione centrale del Ministero degli affari esteri con il decreto del Presidente della Repubblica 19 dicembre 2007, n. 258 . . . . Pag. 6

Ministero dell'economia e delle finanze

DECRETO 10 marzo 2008.

Accertamento del tasso d'interesse semestrale dei certificati di credito del Tesoro 1° settembre 2001/2008, 1° marzo 2005/2012 e 1° marzo 2007/2014, relativamente alle semestralità con decorrenza 1° marzo 2008 e scadenza 1° settembre 2008. Pag. 17

DECRETO 19 marzo 2008.

Riapertura delle operazioni di sottoscrizione dei certificati di credito del Tesoro «zero coupon», con decorrenza 2 gennaio 2008 e scadenza 31 dicembre 2009, settima e ottava tranche. Pag. 17

DECRETO 19 marzo 2008.

Riapertura delle operazioni di sottoscrizione dei buoni del Tesoro poliennali 1,85%, indicizzati all'inflazione europea, con godimento 15 marzo 2007 e scadenza 15 settembre 2012, nona e decima tranche . . . . Pag. 19

DECRETO 19 marzo 2008.

Riapertura delle operazioni di sottoscrizione dei buoni del Tesoro poliennali 2,35%, con godimento 15 settembre 2004 e scadenza 15 settembre 2035, diciannovesima e ventesima tranche, indicizzati all'inflazione europea . . . . Pag. 21

DECRETO 20 marzo 2008.

Fondazioni bancarie. Misure dell'accantonamento alla riserva obbligatoria e dell'accantonamento patrimoniale facoltativo per l'esercizio 2007 . . . . Pag. 23

DECRETO 20 marzo 2008.

Modalità tecniche di svolgimento della lotteria nazionale «Giornata del bambino africano del Programma alimentare mondiale», «Gran premio di Agnano» e «Lotteria del consumatore» - Manifestazione 2008 . . . . Pag. 24

Ministero della salute

DECRETO 22 gennaio 2008.

Revoca dei decreti del Ministro della salute del 16 ottobre 2003 e del 20 aprile 2006 di attuazione della decisione della Commissione 2003/308/CE del 2 maggio 2003 — relativa alla non iscrizione della sostanza attiva metalaxil nell'allegato I della direttiva 91/414/CEE — e modifica dell'allegato al decreto del Ministro della salute del 20 aprile 2006 Pag. 28

DECRETO 8 febbraio 2008.

Autorizzazione all'immissione in commercio del prodotto fitosanitario «Vincare», registrato al n. 11948 . . . . . Pag. 31

DECRETO 20 febbraio 2008.

Autorizzazione all'immissione in commercio del prodotto fitosanitario «Kelion 50 WG», registrato al n. 12885 Pag. 34

DECRETO 7 marzo 2008.

Elenco dei prodotti fitosanitari contenenti la sostanza carbendazim, revocati ai sensi dell'articolo 2, comma 4, del decreto del Ministero della salute 17 ottobre 2007, relativo all'iscrizione della sostanza attiva carbendazim nell'allegato I del decreto legislativo 17 marzo 1995, n. 194. . . Pag. 36

DECRETO 14 marzo 2008.

Sospensione della validità del decreto di riconoscimento dell'acqua minerale «Mitica», in Fonni . . . . Pag. 37

DECRETO 14 marzo 2008.

Sospensione della validità del decreto di riconoscimento dell'acqua minerale «Grazia», in Corigliano d'Otranto. Pag. 37

DECRETO 14 marzo 2008.

Sospensione della validità dei decreti di riconoscimento delle acque minerali «Aemilia» e «Madonna della Mercede», in Medesano. . . . . Pag. 38

DECRETO 14 marzo 2008.

Sospensione della validità dei decreti di riconoscimento delle acque minerali «Coralba» e «Font dei Signuri», in San Damiano Macra. . . . . Pag. 38

DECRETO 14 marzo 2008.

Sospensione della validità del decreto di riconoscimento dell'acqua minerale «Il Bric», in Rorà . . . . Pag. 39

DECRETO 17 marzo 2008.

Non iscrizione della sostanza attiva metomil nell'allegato I del decreto legislativo 17 marzo 1995, n. 194, e revoca dei prodotti fitosanitari contenenti detta sostanza attiva . . . Pag. 39

Ministero delle politiche agricole alimentari e forestali

DECRETO 24 gennaio 2008.

Recepimento della decisione della Commissione U.E. n. 2007/842/CE del 6 dicembre 2007, concernente le misure fitosanitarie d'emergenza contro la propagazione dell'organismo nocivo *Pseudomonas solanacearum* (Smith) Smith per quanto riguarda l'Egitto. . . . . Pag. 41

DECRETO 28 gennaio 2008.

Lotta obbligatoria contro il marciume anulare della patata (*Clavibacter michiganensis* ssp. *Sepedonicus*). Recepimento della direttiva della Commissione 2006/56/CE. . . . . Pag. 43

DECRETO 28 gennaio 2008.

Attuazione della decisione della Commissione U.E. n. 2007/410/CE del 12 giugno 2007, relativa alle misure per impedire l'introduzione e la diffusione all'interno della Comunità del viroide dell'affusolamento dei tuberi di patata . . . . . Pag. 45

DECRETO 26 febbraio 2008.

Proroga dei termini di cui all'articolo 6 del decreto 31 gennaio 2008, n. 1229, concernente «Disposizioni nazionali di attuazione del regime transitorio di cui all'articolo 68-*ter* del regolamento (CE) n. 1782/2003, previsto dalla riforma della politica agricola comune nel settore del pomodoro destinato alla trasformazione» in ordine alla stipula e al deposito dei contratti di trasformazione del pomodoro per l'anno 2008 Pag. 47

DECRETO 27 febbraio 2008.

Modifica dell'articolo 4 del decreto 22 ottobre 2007, n. 1540, concernente «Disposizioni per l'attuazione della riforma della politica agricola comune nel settore del pomodoro destinato alla trasformazione» in ordine al termine di comunicazione dei casi di forza maggiore o di circostanze eccezionali . . . . Pag. 47

DECRETO 29 febbraio 2008.

Disposizioni nazionali di attuazione del regime transitorio di cui all'articolo 68-*ter* del regolamento (CE) n. 1782/2003 previsto dalla riforma della politica agricola comune nel settore delle pere, delle pesche e delle prugne d'Ente destinate alla trasformazione. . . . . Pag. 48

DECRETO 12 marzo 2008.

Iscrizione della varietà di patata «Chopin» al registro nazionale delle varietà di specie agrarie . . . . Pag. 52

DECRETO 12 marzo 2008.

Iscrizione della varietà di lino «Antello» al registro nazionale delle varietà di specie agrarie . . . . Pag. 52

DECRETO 17 marzo 2008.

Disposizioni inerenti la ripartizione tra le regioni e le province autonome delle risorse finanziarie e degli ettari oggetto del regime di ristrutturazione e riconversione dei vigneti per la campagna 2007/2008 di cui al regolamento CE n. 1493/99. Pag. 53

DECRETO 20 marzo 2008.

Disposizioni in materia di violazioni riscontrate nell'ambito del regolamento (CE) n. 1782/03 del Consiglio, del 29 settembre 2003 sulla PAC e del regolamento (CE) 1698/2005 del Consiglio, del 20 settembre 2005, sul sostegno allo sviluppo rurale da parte del Fondo europeo agricolo per lo sviluppo rurale (FEASR) . . . . Pag. 56

Ministero dei trasporti

DECRETO 11 gennaio 2008.

Recepimento della direttiva 2007/38/CE del Parlamento europeo e del Consiglio, dell'11 luglio 2007, concernente l'installazione a posteriori di specchi sui veicoli commerciali pesanti immatricolati nella Comunità. . . . . Pag. 64

DECRETI E DELIBERE DI ALTRE AUTORITÀ

Comitato interministeriale per la programmazione economica

DELIBERAZIONE 23 novembre 2007.

Direttive per la semplificazione dei criteri di riparto e di gestione del cofinanziamento nazionale dei progetti strategici di cui all'articolo 16, comma 1, della legge 7 agosto 1997, n. 266. (Deliberazione n. 125/2007). . . . . Pag. 65

Agenzia italiana del farmaco

DETERMINAZIONE 20 marzo 2008.

Linee guida per la classificazione e conduzione degli studi osservazionali sui farmaci. . . . . Pag. 68

ESTRATTI, SUNTI E COMUNICATI

Ministero dell'interno:

Riconoscimento e classificazione di alcuni prodotti esplosivi. . . . . Pag. 75

Riconoscimento della personalità giuridica al Pontificio Ateneo S. Anselmo, in Roma . . . . Pag. 86

Riconoscimento della personalità giuridica alla Casa Suore dell'Immacolata, in Genova . . . . Pag. 86

Approvazione del trasferimento di sede dell'Istituto delle Figlie del Sacro Cuore di Gesù, in Roma . . . . Pag. 86

Ministero della salute:

Autorizzazione all'immissione in commercio, mediante procedura di mutuo riconoscimento, del medicinale per uso veterinario «Fatromectin 10 mg/ml». . . . . Pag. 86

Autorizzazione all'immissione in commercio, mediante procedura di mutuo riconoscimento, del medicinale per uso veterinario ad azione immunologica «Hiprabovis Pneumos». Pag. 87

Modificazione dell'autorizzazione all'immissione in commercio del medicinale per uso veterinario «Acqua per preparazioni iniettabili Panpharma» . . . . Pag. 87

Modificazione dell'autorizzazione all'immissione in commercio del medicinale per uso veterinario «Elettrolitica reidratante III Panpharma». . . . . Pag. 87

Modificazione dell'autorizzazione all'immissione in commercio del medicinale per uso veterinario «Calcio gluconato 20% Panpharma». . . . . Pag. 88

Modificazione dell'autorizzazione all'immissione in commercio del medicinale per uso veterinario «Tinkanium». Pag. 88

Modificazione dell'autorizzazione all'immissione in commercio del medicinale per uso veterinario «Meflosyl» Pag. 88

Modificazione dell'autorizzazione all'immissione in commercio del medicinale veterinario «Izochinossal» . . Pag. 88

Modificazione dell'autorizzazione all'immissione in commercio del medicinale per uso veterinario «Stimulfos». Pag. 88

Ministero delle politiche agricole alimentari e forestali: Proposta di modifica del disciplinare di produzione della denominazione di origine protetta «Pane di Altamura» . . . Pag. 89

Ministero dello sviluppo economico:

Sospensione a tempo indeterminato dell'autorizzazione all'esercizio dell'attività fiduciaria e di revisione, rilasciata alla società «Danubio fiduciaria e di revisione in liquidazione - S.r.l.», in Milano . . . . Pag. 91

Autorizzazione ad esercitare un magazzino generale in Trieste . . . . Pag. 91

Agenzia italiana del farmaco: Comunicato di rettifica relativo all'estratto della determinazione n. 732/2007 del 18 dicembre 2007, recante l'autorizzazione all'immissione in commercio del medicinale per uso umano «Ramipril Arrow». Pag. 91

Cassa depositi e prestiti S.p.a.: Avviso relativo all'emissione di nuove serie di buoni fruttiferi postali contraddistinte con le sigle «B42», «28F», «1F8», «I27», «M22» e «P07». Pag. 91

RETTIFICHE

ERRATA-CORRIGE

Avviso relativo al comunicato del Ministero dei trasporti, recante: «Passaggio dal demanio al patrimonio dello Stato di un immobile nel comune di Albenga.». . . . . Pag. 92

SUPPLEMENTI STRAORDINARI

MINISTERO DELL'ECONOMIA
E DELLE FINANZE

Decreti recanti l'approvazione di 68 studi di settore relativi ad attività economiche nel settore delle manifatture, dei servizi, del commercio e ad attività professionali e l'aggiornamento dello studio della territorialità.

08A02075

8. *Disposizioni attuative.*

Con il decreto ministeriale di cui al precedente punto 3.3. verranno individuate le priorità cui i progetti strategici dovranno ispirarsi, le modalità di presentazione degli stessi, i soggetti beneficiari degli interventi regionali, le spese agevolabili, nonché le ulteriori disposizioni di attuazione della presente deliberazione.

Roma, 23 novembre 2007

Il Presidente: PRODI

Il segretario del CIPE: GOBBO

Registrato alla Corte dei conti il 18 marzo 2008
Ufficio controllo atti Ministeri economico-finanziari, registro n. 1 Economia e finanze, foglio n. 350

08A02148

AGENZIA ITALIANA DEL FARMACO

DETERMINAZIONE 20 marzo 2008.

Linee guida per la classificazione e conduzione degli studi osservazionali sui farmaci.

IL DIRETTORE GENERALE

Visti gli articoli 8 e 9 del decreto legislativo 30 luglio 1999, n. 300;

Visto l'art. 48 del decreto-legge 30 settembre 2003, n. 269, convertito nella legge 24 novembre 2003, n. 326, che istituisce l'Agenzia italiana del farmaco ed in particolare il comma 13;

Visto il decreto del Ministro della salute di concerto con i Ministri della funzione pubblica e dell'economia e finanze in data 20 settembre 2004, n. 245, recante norme sull'organizzazione ed il funzionamento dell'Agenzia italiana del farmaco, a norma del comma 13 dell'art. 48 sopra citato, ed in particolare l'art. 19;

Visto il decreto del Ministero della salute 12 maggio 2006 che istituisce e regola il funzionamento dei Comitati etici;

Vista la direttiva 2001/20/CE relativa all'applicazione della buona pratica clinica nell'esecuzione delle sperimentazioni cliniche di medicinali per uso clinico;

Visto il decreto legislativo 24 giugno 2003, n. 211 «Attuazione della direttiva 2001/20/CE relativa all'applicazione della buona pratica clinica nell'esecuzione delle sperimentazioni cliniche di medicinali per uso clinico», e successive modifiche e integrazioni;

Vista la circolare del Ministero della salute del 2 settembre 2002, che definisce, in linea generale, i criteri per la valutazione degli studi clinici osservazionali (o non sperimentali o non interventistici), di seguito indicati come studi osservazionali;

Rilevato che un numero crescente di studi definiti osservazionali il cui obiettivo implica la valutazione di uno o più farmaci viene sottoposto all'attenzione dei comitati etici;

Considerata la scarsità e la genericità delle nozioni riguardanti i vari aspetti degli studi osservazionali presenti nella normativa di riferimento innanzi citata e la conseguente incertezza circa l'opportunità e le modalità di valutazione dei menzionati studi da parte dei comitati etici;

Considerato che gli studi osservazionali rivestono particolare importanza per valutazioni epidemiologiche, farmacoepidemiologiche e di farmacosorveglianza e possono essere utilizzati anche per stime economiche, di qualità, prescrittive e di carichi assistenziali, nonché per la valutazione del profilo di sicurezza di farmaci, nelle normali condizioni di uso, su grandi numeri di soggetti;

Rilevato che, conseguentemente, gli studi osservazionali hanno particolare rilevanza per l'analisi della qualità delle cure, dei profili assistenziali e delle relative ricadute economiche;

Ritenuto opportuno approvare il documento «Linee guida sugli studi osservazionali», di cui all'allegato 1, parte integrante e sostanziale del presente decreto, al fine di consentire una valutazione omogenea dei protocolli relativi agli studi medesimi da parte dei comitati etici;

Rilevata la necessità di istituire un registro nazionale degli studi osservazionali;

Visto il decreto del Ministro della salute 30 aprile 2004 di nomina del dott. Nello Martini in qualità di direttore generale dell'Agenzia italiana del farmaco, registrato in data 17 giugno 2004 al n. 1154 del registro visti semplici dell'Ufficio centrale di bilancio presso il Ministero della salute;

Visto il decreto del Ministro della salute 28 settembre 2004 che ha costituito la Commissione consultiva tecnico-scientifica dell'Agenzia italiana del farmaco;

Determina:

Art. 1.

Sono approvate le linee guida sugli studi osservazionali di cui all'allegato 1 e alle appendici 1 e 2, parte integrante e sostanziale del presente atto, al fine di consentire una valutazione omogenea dei protocolli relativi agli studi medesimi da parte dei comitati etici.

Art. 2.

È istituito il registro nazionale degli studi osservazionali, al fine di effettuare analisi descrittive e di predisporre report periodici; i contenuti e le modalità operative di funzionamento ed utilizzo del registro saranno comunicate agli operatori tramite il sito internet dell'AIFA.

La presente determinazione ha effetto dal giorno successivo alla sua pubblicazione nella *Gazzetta Ufficiale* della Repubblica italiana.

Roma, 20 marzo 2008

Il direttore generale: MARTINI

ITALIAN DRUG AGENCY
DETERMINATION March 20, 2008.

Guidelines for the classification and conduct of observational drug studies.

THE GENERAL DIRECTOR

Considering the articles 8 and 9 of the legislative decree 30 July 1999, n. 300;

Given the art. 48 of the decree-law 30 September 2003, n. 269, converted into law no. 326, which establishes the Italian Medicines Agency and in particular paragraph 13;

Having regard to the decree of the Minister of Health in agreement with the Ministers of the public function and of the economy and finance dated 20 September 2004, n. 245, laying down rules on the organization and functioning of the Italian Medicines Agency, pursuant to paragraph 13 of art. 48 mentioned above, and in particular art. 19;

Having regard to the decree of the Ministry of Health of 12 May 2006 which establishes and regulates the functioning of the Ethics Committees;

Having regard to Directive 2001/20 / EC on the application of good clinical practice in the execution of clinical trials of medicinal products for clinical use;

Given the legislative decree of 24 June 2003, n. 211 "Implementation of Directive 2001/20 / EC relating to the application of good clinical practice in the execution of clinical trials of medicinal products for clinical use", and subsequent amendments and additions;

Having regard to the circular of the Ministry of Health of 2 September 2002, which generally defines the criteria for the evaluation of observational (or non-experimental or non-interventional) clinical studies, hereinafter referred to as observational studies;

It has been noted that an increasing number of so-called observational studies whose objective involves the evaluation of one or more drugs are submitted to the attention of the ethics committees;

Considering the scarcity and generality of the notions concerning the various aspects of observational studies present in the reference legislation cited above and the consequent uncertainty about the opportunity and methods of evaluation of the aforementioned studies by the ethics committees;

Considering that observational studies are of particular importance for epidemiological, pharmacoepidemiological and pharmacosurveillance evaluations and can also be used for economic, quality, prescriptive and care burden estimates, as well as. for the evaluation of the safety profile of drugs, under normal conditions of use, on large numbers of subjects;

Having noted that, consequently, the observational studies have particular relevance for the analysis of the quality of care, of the assistance profiles and of the related economic repercussions;

It was deemed appropriate to approve the document "Guidelines on observational studies", referred to in Annex 1, an integral and substantial part of this decree, in order to allow a homogeneous evaluation of the protocols relating to the studies themselves by the ethics committees;

The need to establish a national register of observational studies has been identified;

Given the decree of the Minister of Health April 30, 2004 appointing Dr. Nello Martini as general manager of the Italian Medicines Agency, registered on June 17, 2004 under no. 1154 of the simple visa register of the Central Budget Office at the Ministry of Health;

Given the decree of the Minister of Health of 28 September 2004 which set up the Advisory Commission technical-scientific of the Italian Medicines Agency;

Determine:

Art. 1.

The guidelines on observational studies referred to in Annex 1 and Appendices 1 and 2, an integral and substantial part of this document, are approved in order to allow a homogeneous evaluation of the protocols relating to the same studies by the ethics committees.

Art. 2.

The national register of observational studies is established in order to carry out descriptive analyzes and prepare periodic reports; the contents and operating methods of operation and use of the register will be communicated to operators through the AIFA website.

This determination takes effect from the day following its publication in the Official Gazette of the Italian Republic.

Rome, March 20, 2008

The General Director: MARTINI

ALLEGATO 1

Linee guida per gli studi osservazionali sui farmaci

1. Premessa

In tutti i Paesi sono adottate regole e normative finalizzate a tutelare i soggetti coinvolti nelle sperimentazioni cliniche di tipo interventistico. In Italia questo settore è regolamentato soprattutto dal decreto legislativo 211/2003 *"Attuazione della Direttiva 2001/20/CE del Parlamento Europeo e del Consiglio relativa all'applicazione della buona pratica clinica nell'esecuzione delle sperimentazioni cliniche di medicinali ad uso umano"*.

L'Italia ha inoltre adottato una specifica normativa sui Comitati etici (DM 12/5/2006) attraverso cui ha affidato loro l'emanazione del parere sulle ricerche cliniche.
Infine, da Settembre 2002 è presente una regolamentazione riguardante i criteri per la valutazione degli studi clinici osservazionali (o non sperimentali o non interventistici) (Circolare 6 del 2/9/2002).

Gli studi osservazionali sui farmaci sono di particolare importanza per la valutazione del profilo di sicurezza nelle normali condizioni di uso e su grandi numeri di soggetti, per approfondimenti sull'efficacia nella pratica clinica, per la verifica dell'appropriatezza prescrittiva e per valutazioni di tipo farmacoeconomico.

Per le loro caratteristiche, gli studi osservazionali non comportano rischi aggiuntivi per i soggetti ai quali sono offerte le migliori condizioni di assistenza clinica. Di conseguenza richiedono procedure differenziate rispetto a quanto previsto negli studi clinici sperimentali.

Una particolare cautela è richiesta per evitare che una sperimentazione sia presentata come uno studio osservazionale.

A questo proposito va ricordato che, in coerenza con quanto previsto dal decreto legislativo 211/2003, per poter essere considerati osservazionali gli studi riguardanti un farmaco devono soddisfare le seguenti condizioni:

1. Il farmaco deve essere prescritto nelle indicazioni d'uso autorizzate all'immissione in commercio in Italia;
2. La prescrizione del farmaco in esame deve essere parte della normale pratica clinica;
3. La decisione di prescrivere il farmaco al singolo soggetto deve essere del tutto indipendente da quella di includere il soggetto stesso nello studio (ove applicabile);
4. Le procedure diagnostiche e valutative devono corrispondere alla pratica clinica corrente

Si ritiene indispensabile che i Comitati etici siano informati sullo svolgimento di questi studi nella struttura o sul territorio di loro pertinenza. E' quindi necessario che, a seconda dello studio osservazionale proposto i Comitati etici ricevano sempre una notifica dello studio oppure una richiesta formale per la formulazione di un parere.

2. Protocollo

Ogni Studio osservazionale deve fondarsi su un protocollo in cui gli obiettivi ed il disegno dello studio devono essere definiti in modo chiaro e coerente. Nel protocollo presentato deve essere chiaramente valutabile l'ipotesi della ricerca, i risultati attesi, il tipo di studio osservazionale, la

Guidelines for Observational Drug Studies

1. Introduction

In all countries, rules and regulations are adopted aimed at protecting those involved in interventional clinical trials. In Italy this sector is regulated above all by the legislative decree 211/2003 "Implementation of Directive 2001/20/EC of the European Parliament and of the Council concerning the application of good clinical practice in the execution of clinical trials of medicinal products for human use."

Italy has also adopted specific legislation on ethics committees (Ministerial Decree 12/5/2006) through which it has entrusted them with the issue of the opinion on clinical research.

Finally, since September 2002 there has been a regulation concerning the criteria for the evaluation of observational clinical studies (either non-experimental or non-interventional) (Circular 6 of 2/9/2002).

Observational studies on drugs are of particular importance for the evaluation of the safety profile in normal conditions of use and on large numbers of subjects, for further information on efficacy in clinical practice, for the verification of prescribing appropriateness and for pharmacoeconomic evaluations.

Due to their characteristics, observational studies do not involve additional risks for those who are offered the best conditions of clinical care. Consequently, they require different procedures than those envisaged in experimental clinical studies.

Particular caution is required to prevent a trial from being presented as an observational study.

In this regard, it should be remembere that, in accordance with the provisions of Legislative Decree 211/2003, in order to be considered observational, studies concerning a drug must meet the following conditions:

1. The drug must be prescribed in the indications for use authorized for marketing in Italy;
2. The prescription of the drug in question must be part of normal clinical practice;
3. The decision to prescribe the drug to an individual subject must be completely independent from that of including the subject in the study (where applicable);
4. Diagnostic and evaluation procedures should correspond to current clinical practice

It is considered essential that the ethics committees are informed about the conduct of these studies in the structure or in the territory of their pertinence. It is therefore necessary that, depending on the observational study proposed, the Ethics Committees always receive a notification of the study or a formal request for the formulation of an opinion.

2. Protocol

Each observational study must be based on a protocol in which the objectives and design of the study must be clearly and coherently defined. In the protocol presented, the hypothesis of the research, the expected results, the type of observational study, the

scelta della dimensione campionaria, le informazioni che saranno raccolte, l'eventuale coinvolgimento della struttura e/o degli operatori sanitari, le risorse richieste, l'origine del finanziamento, le modalità di partecipazione e di informazione rivolte al soggetto. Modifiche sostanziali al protocollo dello studio dovranno essere notificate ai Comitati etici secondo quanto previsto per quella specifica tipologia di studio.

3. Segnalazioni Reazioni Avverse

Le reazioni avverse dovranno essere segnalate analogamente a quanto previsto dalle norme in vigore per le segnalazioni spontanee (post-marketing).

4. Siti presso i quali si effettuano gli studi

Gli studi osservazionali possono essere condotti presso le strutture sanitarie pubbliche (o ad esse equiparate), presso le strutture sanitarie private, presso i Medici di Medicina Generale e/o Pediatri di Libera Scelta, presso i Medici che svolgono attività libero-professionale, facendo riferimento per le notifiche e le approvazioni allo specifico Comitato etico competente per il territorio. Nel caso dei Medici di Medicina Generale e/o Pediatri di Libera Scelta non si prevede la necessità che tali operatori, per poter condurre gli studi oggetto delle presenti linee guida, siano iscritti negli appositi registri previsti dal DM 10/5/2001 "Sperimentazione clinica controllata in medicina generale e pediatria di libera scelta")

5. Aspetti Economici

Gli eventuali compensi previsti per gli operatori coinvolti devono essere notificati al Comitato etico insieme alla documentazione dello studio. Nel caso di operatori afferenti a strutture pubbliche o vincolati da rapporto pubblico con le ASL, l'erogazione delle somme deve sempre avvenire per il tramite dell'Ente di appartenenza. E' importante che tali compensi siano commisurati all'effettivo impegno richiesto alla struttura e comunque di valore tale da non influenzare l'operato del personale sanitario coinvolto.

6. Copertura assicurativa

Data la natura osservazionale degli studi proposti, non sono necessarie polizze assicurative aggiuntive rispetto a quelle già previste per la normale pratica clinica.

7. Pubblicazione dei risultati

In funzione di quanto già previsto dalla circolare di Settembre 2002, in fase di presentazione dello studio deve essere esplicito e scritto l'impegno da parte del proponente alla stesura di un rapporto finale e a rendere pubblici i risultati al termine dello studio.

8. Registro degli studi osservazionali

Viene istituito presso l'Agenzia Italiana del Farmaco il Registro Nazionale studi osservazionali al quale dovranno essere inviati i dati relativi agli studi in modo esclusivamente telematico.

9. Implicazioni operative per i proponenti e i Comitati etici

Si individua di seguito la documentazione che i proponenti devono allegare per la presentazione degli studi ai Comitati etici:

- Dichiarazione sulla natura osservazionale dello studio (appendice 1);
    - Per studio promosso da azienda privata (società di capitali), a firma del rappresentate del promotore e dello Sperimentatore Coordinatore (in caso di studio multicentrico) o del Responsabile dello studio (studio monocentrico)
    - Per studio non promosso da azienda privata (società di capitali), a firma dello Sperimentatore Coordinatore (in caso di studio multicentrico) o del Responsabile dello studio (studio monocentrico)

choice of the sample size, the information that will be collected, the possible involvement of the structure and/or health professionals, the resources requested, the origin of the financing, the methods of participation and information addressed to the subject. Substantial changes to the study protocol must be notified to the Ethics Committees in accordance with the provisions for that specific type of study.

3. Reporting Adverse Reactions
Adverse reactions must be reported in the same way as required by the regulations in force for spontaneous reporting (post-marketing).

4. Sites where studies are carried out
Observational studies can be conducted at public health facilities (or equivalent to them), at private health facilities, at General Practitioners and/or Free Choice Pediatricians, at Physicians who carry out freelance activities, referring to for notifications and approvals to the specific Ethics Committee competent for the territory. In the case of General Practitioners and/or Free Choice Pediatricians, it is not envisaged that these operators, in order to conduct the studies covered by these guidelines, are registered in the appropriate registers provided for by the Ministerial Decre 10/5/2001 “Clinical trial controlled in general medicine and free choice pediatrics”)

5. Economic Aspects
Any remuneration envisaged for the operators involved must be notified to the Ethics Committee together with the documentation of the study. In the case of operators belonging to public structures or bound by a public relationship with the ASL, the disbursement of the sums must always take place through the institution to which they belong. It is important that these fees are commensurate with the actual commitment required of the structure and in any case of such value as not to influence the work of the health personnel involved.

6. Insurance coverage
Given the observational nature of the proposed studies, no additional insurance policies are required to those already envisaged for normal clinical practice.

7. Publication of results
In accordance with the provisions of the circular of September 2002, when submitting the study, the proposer's commitment to draft a final report and to make the results public at the end of the study must be explicit and written.

8. Register of observational studies
The National Observational Studies Register is set up at the Italian Medicines Agency to which the data relating to the studies must be sent exclusively electronically.

9. Operational implications for proponents and ethics committees
Below is the documentation that proposers must attach for the submission of studies to the Ethics Committees:

Statement on the observational nature ofthe study (Appendix 1);
- For study promoted by a private company (joint stock company), signed by the representative of the promoter and the Coordinating Investigator (in the case of a multicentre study) or by the Head of the study (monocentric study)
- For study not promoted by a private company (capital venture), signed by the Investigator Coordinator (in case of a multicenter study) or of the Head of the study (single center study)

- Protocollo;
- Elenco delle informazioni che si vogliono raccogliere;
- Dettagli riguardanti il responsabile e la sede in cui si svolgerà lo studio, al fine di consentire eventuali accertamenti ispettivi;
- Lista dei centri partecipanti e relativi responsabili (nell'ipotesi di studi multicentrici)
- Eventuali costi aggiuntivi derivanti dalla conduzione dello studio (e relativa copertura);
- Identificazione delle fonti di finanziamento;
- Nota informativa al soggetto e modulo di consenso al trattamento dei dati personali (nel casi di studi che prevedano un rapporto diretto con il soggetto);
- Descrizione delle procedure messe in atto per garantire la confidenzialità delle informazioni;
- Proposta di convenzione con riferimento particolare agli aspetti finanziari (se previsto);
- Eventuali compensi previsti per il responsabile dello studio nonché per gli sperimentatori coinvolti;
- Lettera informativa al medico curante (ove applicabile).

10. Procedure generali per l'avvio degli studi osservazionali

Per gli studi di coorte prospettici nei quali i soggetti sono inclusi nello studio in base all'assunzione di un determinato farmaco (o di una combinazione approvata di essi) e seguiti nel tempo per la valutazione degli esiti, deve essere sempre richiesta la formale approvazione al Comitato etico.

In questa tipologia, nel caso di studio multicentrico, il proponente dovrà individuare tra i Comitati etici dei centri partecipanti quello che avrà le funzioni di coordinamento (in cui opererà lo Sperimentatore coordinatore). Tale Comitato etico avrà il compito di rilasciare al proponente, per iscritto entro 45 giorni dalla data di presentazione, il parere unico sullo studio.
Contestualmente il proponente potrà sottomettere anche agli altri Comitati di Etica la documentazione dello studio prevista nell'appendice 2 al fine di consentire loro, se del caso, di inviare eventuali osservazioni al Comitato etico coordinatore.

Tali Comitati etici avranno poi il compito di rilasciare al Proponente, per iscritto ed entro 30 giorni dalla ricezione del parere unico, formale accettazione o motivato rifiuto per la partecipazione allo studio.

Nel caso di Parere unico negativo il proponente avrà la facoltà di rivedere il protocollo e riproporlo allo stesso Comitato etico. Nel caso di un secondo parere unico negativo lo studio non potrà essere condotto in Italia.

Per quanto concerne le altre tipologie di studi osservazionali sarà sufficiente, da parte del proponente, la notifica ai Comitati etici dei centri partecipanti; in seguito lo studio potrà iniziare dopo 60 giorni dalla data di notifica utilizzando la procedura del silenzio/assenso.
Resta inteso che tutti i Comitati etici hanno comunque facoltà di valutare tutti gli aspetti degli studi (anche solo notificati) in accordo alle proprie procedure interne segnalando, ove rilevato, incongruità rispetto a quanto dichiarato dal promotore dello studio.

Indipendentemente dalla tipologia dello studio, valgono per tutti gli studi osservazionali i principi generali e le regole vigenti che riguardano il trattamento dei dati personali.

- Protocol;
- List of information to be collected;
- Details regarding the manager and the location where the study will take place, in order to allow any inspections;
- List of participating centers and their managers (in the hypothesis of multicentre studies)
- Any additional costs deriving from the conduct of the study (and related coverage);
- Identification of funding sources;
- Information note to the subject and consent form for the processing of personal data (in the case of studies that envisage a direct relationship with the subject);
- Description of the procedures put in place to guarantee the confidentiality of information;
- Proposal for an agreement with particular reference to the financial aspects (if applicable);
- Any fees provided for the head of the study as well as for the investigators involved;
- Letter of information to the attending physician (where applicable).

10. General procedures for starting observational studies

For prospective cohort studies in which subjects are included in the study on the basis of taking a certain drug (or an approved combination thereof) and followed up over time for the evaluation of outcomes, formal approval must always be requested from the Ethics Committee.

In this typology, in the case of multicentre study, the proposer will have to identify among the ethics committees of the participating centers the one that will have the coordination functions (in which the coordinating Investigator will operate). This Ethics Committee will have the task of issuing to the proposer, in writing within 45 days from the date of submission, the single opinion on the study.

At the same time, the proposer may also submit to the other Ethics Committees the documentation of the study provided for in Appendix 2 in order to allow them, if necessary, to send any observations to the coordinating Ethics Committee.

These Ethics Committees will then have the task of issuing to the Proponent, in writing and within 30 days of receipt of the single opinion, formal acceptance or motivated refusal for participation in the study.

In the case of a single negative opinion, the proposer will have the right to review the protocol and propose it again to the same Ethics Committee. In the event of a second negative single opinion, the study cannot be conducted in Italy.

As regards the other types of observational studies, it will be sufficient for the proposer to notify the ethics committees of the participating centers; thereafter the study can start after SO days from the date of notification using the silence/consent procedure.

It is understood that all the ethics committees have the right to evaluate all aspects of the studies (even if only notified) in accordance with their internal procedures, reporting, where detected, inconsistencies with respect to what was declared by the promoter of the study.

Regardless of the type of study, the general principles and rules in force concerning the processing of personal data apply to all observational studies.

Tabella 1: Tipologia di studi osservazionali

1) studi di coorte prospettici
2) altri studi osservazionali<br>a) studi di coorte retrospettivi<br>b) studi caso-controllo<br>c) studi solo su casi ("*case cross-over*" e "*case series*")<br>d) studi trasversali<br>e) studi di appropriatezza

In ciascuno degli studi indicati possono essere anche presenti obiettivi di valutazione economica dell'uso dei farmaci (farmacoeconomia).

Table 1: Type of observational studies

1. prospective cohort studies
2. other observational studies
   a. retrospective cohort studies
   b. case-control studies
   c. case studies only ("case cross-over"and"case series")
   d. cross-sectional studies
   e. appropriateness studies

In each of the studies indicated there may also be objectives for the economic evaluation of the use of drug (pharmacoeconomics).

APPENDICE 1

DICHIARAZIONE SULLA NATURA OSSERVAZIONALE DELLO STUDIO

Coordinatore/Responsabile dello Studio Osservazionale ______________________
(persona fisica che ha il compito di coordinare lo studio) (nome e cognome)

Struttura pubblica o struttura no-profit nella quale opera il Coordinatore

____
(specificare la struttura)

Azienda promotrice dello studio (se diversa da quella già indicata)

____
(specificare l'Azienda)

Io sottoscritto ______________________ In qualità di coordinatore dello studio osservazionale (riportare il titolo)

____

____

____

DICHIARO che:

Il/i farmaco/i è/sono prescritto/i nelle indicazioni d'uso autorizzate all'immissione in commercio in Italia;
La prescrizione del farmaco in esame deve essere parte della normale pratica clinica;
La decisione di prescrivere il farmaco al singolo soggetto è del tutto indipendente da quella di includere il soggetto stesso nello studio (ove applicabile);
Le procedure diagnostiche e valutative corrispondono alla pratica clinica corrente.

Data _____________ Firma del Coordinatore _________________

Firma del Promotore _________________
(ove applicabile)

Si ricorda che per poter considerare uno studio di tipo osservazionale devono essere rispettate congiuntamente tutte e 4 le condizioni sopra riportate

APPENDICE 2

Tavola Riassuntiva

<table>
<tr><th>Tipologia di studi osservazionali</th><th>Copertura Assicurativa</th><th>Notifica al CE</th><th>Richiesta di approvazione al CE</th><th>Documentazione da inviare al CE per tutti gli studi osservazionali</th></tr>
<tr><td>Studi di coorte prospettici</td><td>NO</td><td>NO</td><td>SI</td><td rowspan="2">• Dichiarazione del proponente sulla natura osservazionali dello studio (Appendice 1);
• Protocollo;
• Lista delle informazioni da raccogliere;
• Dettagli riguardanti il responsabile e la sede in cui si svolgerà lo studio, al fine di consentire eventuali accertamenti ispettivi;
• Lista dei centri partecipanti e relativi responsabili (nell'ipotesi di studi multicentrici)
• Eventuali costi aggiuntivi derivanti dalla conduzione dello studio e relativa copertura;
• Identificazione delle fonti di finanziamento;
• Nota informativa al soggetto e modulo di consenso al trattamento dei dati personali *;
• Descrizione delle procedure messe in atto per garantire la confidenzialità delle informazioni;
• Proposta di convenzione con riferimento particolare agli aspetti finanziari (se previsto).
• Eventuali compensi previsti per il responsabile dello studio nonché per gli sperimentatori coinvolti
• Lettera informativa al medico curante (ove applicabile).</td></tr>
<tr><td>Altri studi osservazionali
a) studi di coorte retrospettivi
b) studi caso-controllo
c) studi solo su casi ("case cross-over" e "case series")
d) studi trasversali
e) studi di appropriatezza</td><td>NO</td><td>SI</td><td>NO</td></tr>
</table>

** riguarda solo gli studi nei quali vi sia un rapporto diretto con i soggetti*

Sono da considerare procedure di pratica clinica corrente <u>anche</u>:

1) le visite di follow up purché sostanzialmente corrispondenti alla pratica clinica corrente o a quanto prescritto da linee guida nazionali e/o internazionali;
2) la somministrazione di questionari, interviste, diari, indagini di economia sanitaria e farmacoeconomia, valutazioni soggettive da parte del soggetto sul proprio stato di salute, scale di valutazione ed esami ematochimici*, il cui uso sia giustificato dal razionale dello studio. Non sono considerati studi osservazionali quelli in cui gli esami siano finalizzati a studi di farmacogenetica e/o farmacogenomica.

**Pharmacovigilance, Volume 9a - capitolo 7 parte 1 pag. 87.*

08A02109

45-410100080331

€ 1,00
