# Supplementary material for: Invasive Meningococcal Disease and Meningococcal Serogroup B Vaccination in Adults and Their Offspring: Knowledge, Attitudes, and Practices in Italy (2019)
Source: Vaccines (Basel). 2023 Feb 22;11(3):508. doi: 10.3390/vaccines11030508 (PMC10058645; doi:10.3390/vaccines11030508)
Supplement: Supplementary file 1 [file vaccines-11-00508-s001.zip › SUPPLEMENTARY FILES/MEN - File S3 - questionnaire.docx]

To begin with, some questions about your vaccination status and your children

| Do you live in the Province of Reggio Emilia or Parma? | ( ) YES ( ) NO ( ) N.A. |
| --- | --- |
| Are you born **before** 2002? | ( ) YES ( ) NO ( ) N.A. |
| Do you have any child aged less than 14 years? | ( ) YES ( ) NO ( ) N.A. |

| Have you been vaccinated against Meningococcus (*Neisseria meningigitis*) serogroup C (Men C) | ( ) YES ( ) NO ( ) N.A. |
| --- | --- |
| Have you been vaccinated against Meningococcus (*Neisseria meningigitis*) serogroup B (Men B) | ( ) YES ( ) NO ( ) N.A. |
| Your child(ren) have been vaccinated against Meningococcus (*Neisseria meningigitis*) serogroup C (Men C) | ( ) YES ( ) NO ( ) N.A. |
| Your child(ren) have been vaccinated against Meningococcus (*Neisseria meningigitis*) serogroup B (Men B) | ( ) YES ( ) NO ( ) N.A. |

Let’s speak about your understanding of meningococcal vaccines and natural infection:

| According to your understanding, and regarding its DIFFUSION in the general population, meningitis associated with meningococcus is a … disease | 1 – not significant  (of no significant concern in daily life)  2 – Slightly significant  3 – Somewhat significant  4 – Moderately significant  5 – Very significant  (of very high concern in your daily life) |
| --- | --- |
| According to your understanding, and regarding its SEVERITY in the general population, meningitis associated with meningococcus is a … disease | 1 – not significant  (of no significant concern in daily life)  2 – Slightly significant  3 – Somewhat significant  4 – Moderately significant  5 – Very significant  (of very high concern in your daily life) |
| According to your understanding, meningococcal vaccination is potentially associated with side effects that are … (FREQUENCY) | 1 – not significant  (of no significant concern in daily life)  2 – Slightly significant  3 – Somewhat significant  4 – Moderately significant  5 – Very significant  (of very high concern in your daily life) |
| According to your understanding, menigococcal vaccination is potentially associated with side effects that are … (SEVERITY) | 1 – not significant  (of no significant concern in daily practice)  2 – Slightly significant  3 – Somewhat significant  4 – Moderately significant  5 – Very significant  (of very high concern in your daily life) |

In the following section, we will assess your attitude towards vaccination

| According to your current understanding, regarding meningococcal vaccines (in general) you are | 1 – strongly against  2 – somehow against  3 – neutral  4 – somehow favorable  5 – strongly favorable |
| --- | --- |
| According to your current understanding, regarding vaccination (in general) you are | 1 – strongly against  2 – somehow against  3 – neutral  4 – somehow favorable  5 – strongly favorable |
| According to your personal experiences, how would you rate your CONFIDENCE on the vaccine against Meningococcus B? | 1 – strongly against  2 – somehow against  3 – neutral  4 – somehow favorable  5 – strongly favorable |
| According to your personal experiences, how would you rate your CONFIDENCE on the vaccines, in general? | 1 – strongly against  2 – somehow against  3 – neutral  4 – somehow favorable  5 – strongly favorable |
| According to your personal experiences, how would you rate your CONFIDENCE on the vaccine services? | 1 – strongly against  2 – somehow against  3 – neutral  4 – somehow favorable  5 – strongly favorable |

You have vaccinated your child against MEN B as …

[following section shown only to participants having reported their children was vaccinated with Men B]

| It is included in the National Vaccination Plan | ( ) YES ( ) NO ( ) N.A. |
| --- | --- |
| Suggested by General Practitioner | ( ) YES ( ) NO ( ) N.A. |
| Meningitidis is a severe disease | ( ) YES ( ) NO ( ) N.A. |
| In order to be protected against as many infectious diseases al possible | ( ) YES ( ) NO ( ) N.A. |
| In order to be protected against meningitis | ( ) YES ( ) NO ( ) N.A. |
| A friend has been affected by meningitis | ( ) YES ( ) NO ( ) N.A. |
| In order to avoid bacterial meningitis | ( ) YES ( ) NO ( ) N.A. |
| In order to avoid complications of meningitis | ( ) YES ( ) NO ( ) N.A. |
| In order to avoid transmission of meningitis | ( ) YES ( ) NO ( ) N.A. |
| In order to protect subjects who cannot be vaccinated | ( ) YES ( ) NO ( ) N.A. |

You did **not** vaccinate your child against MEN B as …

[following section shown only to participants having reported their children was not vaccinated with Men B]

| I am against vaccines (in general) | ( ) YES ( ) NO ( ) N.A. |
| --- | --- |
| Preference in other preventive measures | ( ) YES ( ) NO ( ) N.A. |
| Greater trust in antimicrobial treatment | ( ) YES ( ) NO ( ) N.A. |
| Greater trust in alternative therapies e.g. homeopathy | ( ) YES ( ) NO ( ) N.A. |
| Cannot be vaccinated | ( ) YES ( ) NO ( ) N.A. |
| Side Effects after previous vaccination | ( ) YES ( ) NO ( ) N.A. |
| Fear of side effects | ( ) YES ( ) NO ( ) N.A. |
| Lack of trust in “experimental” vaccines | ( ) YES ( ) NO ( ) N.A. |
| Fear of neurological disorders induced by vaccines  (e.g. Guillaume Barré syndrome, multiple sclerosis) | ( ) YES ( ) NO ( ) N.A. |
| Fear of developing autism after vaccine | ( ) YES ( ) NO ( ) N.A. |
| Fear of side effects elicited by vaccine adjuvants | ( ) YES ( ) NO ( ) N.A. |
| Fear of side effects elicited by heavy metals included in the vaccine | ( ) YES ( ) NO ( ) N.A. |
| Not recommended by General Practitioner | ( ) YES ( ) NO ( ) N.A. |
| Aiming to reduce the number of injections | ( ) YES ( ) NO ( ) N.A. |
| Vaccine not affordable | ( ) YES ( ) NO ( ) N.A. |

**Knowledge Test**. In the following section, a total of 18 sentences on healthcare features will be shown to you. Some are true, some are incorrect. Please rate each sentence according to your understanding.

| **Statement** | **Correct Answer** |
| --- | --- |
| Q01. Meningococcus causes around 40-50% of all bacterial meningitis in children aged 2 years or more, and over 70% in children aged 5 years or more. Overall, it causes half of all bacterial meningitis in all infants. | [ ]TRUE  [ ] FALSE  [ ] DON’T KNOW |
| Q02. Mortality of meningococcal meningitis is around 30%, irrespective of therapy | [ ] TRUE  [ ] FALSE  [ ] DON’T KNOW |
| Q03. Early symptoms of meningococcal meningitis are specific, allowing a prompt and appropriate treatment by healthcare providers | [ ] TRUE  [ ] FALSE  [ ] DON’T KNOW |
| Q04. Vaccination against meningococcus reduces spread of bacterium, contributing to herd immunity and ultimately protecting those who cannot be vaccinated | [ ] TRUE  [ ] FALSE  [ ] DON’T KNOW |
| Q05. MenB vaccine may be associated with other vaccinations and vaccine formulates, not increasing the number of accesses to Vaccination Services | [ ] TRUE  [ ] FALSE  [ ] DON’T KNOW |
| Q06. Vaccine additives are not harmful to human beings | [ ] TRUE  [ ] FALSE  [ ] DON’T KNOW |
| Q07. Vaccines against measles may cause severe damages to the central nervous system | [ ] TRUE  [ ] FALSE  [ ] DON’T KNOW |
| Q08. Influenza vaccine may cause severe side effects, potentially lethal | [ ] TRUE  [ ] FALSE  [ ] DON’T KNOW |
| Q09. Measles vaccine may elicit autism | [ ] TRUE  [ ] FALSE  [ ] DON’T KNOW |
| Q10. Some vaccinations may cause diabetes | [ ] TRUE  [ ] FALSE  [ ] DON’T KNOW |
| Q11. Some autoimmune diseases may be elicited by vaccines | [ ] TRUE  [ ] FALSE  [ ] DON’T KNOW |
| Q12. Vaccines are useless, as infectious diseases may be always treated with specific therapies | [ ] TRUE  [ ] FALSE  [ ] DON’T KNOW |
| Q13. Vaccination shots increase probabilities of allergic reactions | [ ] TRUE  [ ] FALSE  [ ] DON’T KNOW |
| Q14. Without vaccinations, smallpox would still exist | [ ] TRUE  [ ] FALSE  [ ] DON’T KNOW |
| Q15. Efficacy of vaccines and vaccination programs has been repetitively proved | [ ] TRUE  [ ] FALSE  [ ] DON’T KNOW |
| Q16. Children would be more resistant to infectious disease without vaccinations | [ ] TRUE  [ ] FALSE  [ ] DON’T KNOW |
| Q17. Many immunizations are performed to early: as a consequence, immune system of children is not allowed to fully develop by itself | [ ] TRUE  [ ] FALSE  [ ] DON’T KNOW |
| Q18. Immune system may be compromised by receiving too many vaccines in pediatric age | [ ] TRUE  [ ] FALSE  [ ] DON’T KNOW |

Finally, some questions about you and your household.

| You are (by your personal understanding): |  |
| --- | --- |
| Male | ( ) |
| Female | ( ) |
| Rather not answer | ( ) |
| Year of birth | ___________ |
| Your higher achievement in terms of education: | ( ) primary school  (< 8 years of formal education)  ( ) secondary school  (8 – 13 years of formal education)  ( ) University or higher |
| You or any of your parents is born abroad? | YES ( )  NO ( )  Prefer no Answer ( ) |
| Do you live with subjects aged 14 years or less? | YES ( )  NO ( )  Prefer no Answer ( ) |
| Do you live with subjects aged 65 years or more? | YES ( )  NO ( )  Prefer no Answer ( ) |
| Size of the household |  |
| No Answer |  |
| Single | ( ) |
| 2 persons | ( ) |
| 3 persons | ( ) |
| 4 persons | ( ) |
| 5 persons or more | ( ) |
| Do you live in… |  |
| Municipality < 5,000 inhabitants | ( ) |
| Municipality 5,000 – 15,000 inhabitants | ( ) |
| Municipality > 15,000 inhabitants | ( ) |
| Main Municipality | ( ) |
| No Answer | ( ) |
| Do you have any background in healthcare settings?  (i.e. have you ever worked in healthcare settings? Have you a formation in healthcare settings?) | ( ) |
