# Supplementary material for: Invasive Meningococcal Disease and Meningococcal Serogroup B Vaccination in Adults and Their Offspring: Knowledge, Attitudes, and Practices in Italy (2019)
Source: Vaccines (Basel). 2023 Feb 22;11(3):508. doi: 10.3390/vaccines11030508 (PMC10058645; doi:10.3390/vaccines11030508)
Supplement: Supplementary file 1 [file vaccines-11-00508-s001.zip › SUPPLEMENTARY FILES/MEN - File S2 - informed consent.docx]

**Supplementary material S2**. Author’s translation of the Informed Consent.

**Informed Consent**. Estimated participant, the present survey has been developed and shared with the aim to assess knowledge, attitudes and practices of the general population regarding the vaccine against serogroup B meningitis. We’re specifically targeting the parents of children aged less than 14 years. The present survey has only scientific aims. No economic or similar compensation are guaranteed to the participants.

While we thank you for your cooperation, we stress that web-based surveys must fulfill the requirements represented by the “Helsinki protocol” and EU Regulation 2016/679.

In order to fulfill the requirements of the Helsinki protocol, we’re requesting to formally share your consent. Without your consent, the survey will not continue. Even after your consent, you can leave the present survey at any moment, until the sharing of the questionnaire (button “share module” at the end of the questionnaire. Moreover, we stress that the questionnaire will be registered in anonymous form, and in no way it could be associated with the compiler, as we will not retain any specific, individual information (e.g., signature, personal address, etc.). All requested personal data are generic ones, and functional to the demographic analyses (gender, age, etc.).

According to the EU Regulation 2016/279 (GDPR), we also state that:

1. data controller, processor, as well as responsible of their retention during the analyses will be Dr. ********** whom you can ask about the process through his personal email (*********). Collected data are generic ones, with SOLE SCIENTIFIC AIMES that have been previously reported. Please be aware that all personal data must be shared with Criminal Law Authorities, without a previous personal consent, in the cases that are specifically reported by the current legal framework, without a specific request, retrieved will not be shared with third parts.
2. after the completion of the questionnaire, we cannot identify in any way the compiler; as the questionnaire is totally anonymous by design, we cannot perform any modification, correction of data collected, and their removal as well.
3. Data will be retained only for the time strictly required for the aforementioned analyses.
